# Integrated renal and sympathetic mechanisms underlying the development of sex- and age-dependent hypertension and the salt sensitivity of blood pressure

**DOI:** 10.1007/s11357-024-01266-1

**Published:** 2024-07-08

**Authors:** Alissa A. Frame, Kayla M. Nist, Kiyoung Kim, Franco Puleo, Jesse D. Moreira, Hailey Swaldi, James McKenna, Richard D. Wainford

**Affiliations:** 1 Department of Pharmacology & Experimental Therapeutics and the Whitaker Cardiovascular Institute, Boston University Chobanian & Avedisian School of Medicine, Boston, MA USA; 2 Department of Anatomy & Neurobiology, Boston University Chobanian & Avedisian School of Medicine, Boston, MA USA; 3 Division of Cardiology, Emory University School of Medicine, 1750 Haygood Drive, Atlanta, GA N22030322 USA; 4 Department of Health Sciences, Sargent College, Boston University, Boston, MA USA

**Keywords:** Aging, Sex differences, Hypertension, Sodium homeostasis, Sodium chloride cotransporter

## Abstract

Aging is a non-modifiable understudied risk factor for hypertension. We hypothesized that sympathetically mediated activation of renal sodium reabsorption drives age-dependent hypertension and the salt sensitivity of blood pressure (BP). Using 3-, 8-, and 16-month-old male and female Sprague–Dawley rats as a model of normal aging, we assessed BP, indices of sympathetic tone, and the physiological responses to acute and chronic sodium challenge including sodium chloride cotransporter (NCC) regulation. The effects of renal nerve ablation and NCC antagonism were assessed in hypertensive male rats. We observed sex-dependent impaired renal sodium handling (24 h sodium balance (meq), male 3-month 0.36 ± 0.1 vs. 16-month 0.84 ± 0.2; sodium load excreted during 5% bodyweight isotonic saline volume expansion (%) male 3-month 77 ± 5 vs. 16-month 22 ± 8), hypertension (MAP (mmHg) male 3-month 123 ± 4 vs. 16-month 148 ± 6), and the salt sensitivity of BP in aged male, but not female, rats. Attenuated sympathoinhibitory afferent renal nerve (ARN) responses contributed to increased sympathetic tone and hypertension in male rats. Increased sympathetic tone contributes to renal sodium retention, in part through increased NCC activity via a dysfunctional with-no-lysine kinase-(WNK) STE20/SPS1-related proline/alanine-rich kinase signaling pathway, to drive hypertension and the salt sensitivity of BP in aged male rats. NCC antagonism and renal nerve ablation, which reduced WNK dysfunction and decreased NCC activity, attenuated age-dependent hypertension in male Sprague–Dawley rats. The contribution of an impaired sympathoinhibitory ARN reflex to sex- and age-dependent hypertension in an NCC-dependent manner, via an impaired WNK1/WNK4 dynamic, suggests this pathway as a mechanism-based target for the treatment of age-dependent hypertension.

**Supplementary Information:**

The online version contains supplementary material available at 10.1007/s11357-024-01266-1.

## Introduction

Hypertension is the leading risk factor for multiple comorbidities, including myocardial infarction, stroke, and chronic kidney disease. The prevalence of hypertension increases with age, from 46% of US adults aged 20–44 years to > 78% of US adults aged over 65 years [1], and the associated risk of hypertension-related morbidity and mortality increases with age [2]. Dietary sodium intake has long been tied to the development of hypertension, as increased sodium retention drives an increase in blood pressure (BP) [3, 4]. Significantly, the salt sensitivity of BP, an exaggerated pressor response to dietary salt intake, which is estimated to impact 50% of hypertensive subjects and 25% of the normotensive population [5], increases with age and profoundly increases the risk of hypertension and adverse cardiovascular outcomes [5, 6]. With 90% of US adults consuming dietary sodium at levels exceeding the National Academies of Science Engineering and Medicine recommended intake of less than 2300 mg sodium/day [7], the lifelong impact of dietary salt intake on BP is a critical public health issue. Despite the wealth of studies examining BP regulation, approximately only 4% of these studies address the role of aging on BP [8]. Therefore, a deeper understanding of the mechanisms influencing sodium homeostasis in age-dependent hypertension may provide new approaches to reduce BP in the elderly.

Sympathetic tone, which contributes to hypertension and the salt sensitivity of BP, increases with age, and positively correlates with elevated BP in adults above the age of 40 [9, 10]. The sympathoinhibitory afferent renal nerve (ARN) reno-renal reflex, which several studies have suggested is integrated in the hypothalamic paraventricular nucleus (PVN) [11, 12], is activated by efferent renal nerve activity and/or mechanical and chemical stimuli [13]. Activation of this sympathoinhibitory reflex suppresses centrally regulated efferent renal nerve activity and renal sodium reabsorption [13]. Highlighting a role of the ARN in BP regulation selective ARN ablation evoked a modest increase in BP in young normotensive male Sprague–Dawley rats [14]. Significantly, removal of all afferent inputs via dorsal rhizotomy and selective removal of the ARN in the young normotensive Sprague–Dawley rat evokes the development of the salt sensitivity of BP [12, 15] and the ARN are involved in DOCA-salt hypertension [16]. Despite evidence for an age-related decrease in markers of the presence of the ARN in aged Sprague–Dawley rats [17], the potential role(s) of the ARN and the sympathoinhibitory ARN reno-reflex in age-related hypertension remain unknown.

Several studies have reported that sympathetic nervous system (SNS)–mediated release of norepinephrine (NE) increases the activity of the sodium chloride cotransporter (NCC) to drive sodium reabsorption at the level of the distal convoluted tubule (DCT) [18, 19] and evokes hypertension. The NCC is regulated by a kinase network that includes with-no-lysine kinases (WNK) 1 and WNK4, STE20/SPS1-related proline alanine rich kinase (SPAK), and oxidative stress response 1 (OxSR1) [20, 21]. The regulatory pathways governing NCC activity are a source of controversy as opposing studies suggest WNK1 or WNK4 as the dominant NCC regulatory kinase [22–24]. There is also debate over which downstream kinase, SPAK or OxSR1, is responsible for phosphorylating and activating the NCC [21]. Further the potential role(s) of the NCC in age-dependent hypertension and age-dependent salt sensitivity of BP remain to be established.

We hypothesized that age-related impairments in the ARN sympathoinhibitory reno-renal reflex contribute to increased sympathetic outflow and subsequent NE-mediated activation of NCC-mediated renal sodium retention to promote age-dependent hypertension and the salt sensitivity of BP in the Sprague–Dawley rat model of normal aging. To test this hypothesis, we assessed acute renal sodium handling, ARN sympathoinhibitory reno-renal reflex activity, BP and sympathetic tone, NCC activity, and NCC regulation in age-dependent hypertension and the impact of high dietary salt intake on these parameters. Collectively, this study provides new mechanistic insight into the sex-dependent sympathetic regulation of NCC-mediated renal sodium handling, via the sympathoinhibitory ARN reno-renal reflex, that influences BP and the salt sensitivity of BP across the lifespan.

## Methods

The data that support the findings of this study are available from the corresponding author upon reasonable request.

### Animals

Male and female Sprague–Dawley rats aged 3, 8, and 16 months old were purchased from Envigo (Indianapolis, IN, USA). Rats were pair-housed prior to surgical intervention or until body weight exceeded 399 g and individual housing was required. All animals, independent of body weight, were individually housed following survival surgery as described below. Animals were housed in a temperature (range 68–79°F) and humidity (range 30–70%) controlled facility under a 12‐h light–dark cycle and were allowed tap water and standard irradiated normal salt rodent diet (NS; Envigo Teklad, WI, Teklad Global Diet #2918, 18% protein, 5% crude fat, 5% fiber, total potassium (K^+^) content 0.6%, total NaCl content 0.6% [174 mEq Na^+^/kg]) or were placed for 21 days on an experimental high-salt diet (HS; Envigo Teklad Diets, WI, TD.03095, 19% protein, 5% crude fat, 3% fiber, total K^+^ content 0.8%, total NaCl content 4% [678 mEq Na^+^/kg]) ad libitum. In all studies, rats were randomly assigned to experimental groups. All animal protocols were approved by the Institutional Animal Care and Use Committee under protocol number PROTO201800201 in accordance with the guidelines of the Boston University Chobanian & Avedisian School of Medicine and the National Institutes of Health *Guide for the Care and Use of Laboratory Animals*. All possible steps were taken to minimize pain and suffering, and euthanasia was conducted in accordance with approved protocols.

### Surgical procedures

#### Acute femoral vein, artery, and bladder cannulation

On day 21 of NS or HS intake, 3-, 8-, and 16-month-old male and female Sprague–Dawley rats were anesthetized (sodium brevital, 20 mg/kg IP, supplemented with 10 mg/kg IV as required) and the left femoral vein, left femoral artery, and bladder were cannulated with PE-50 (vein and artery) or PE-240 (bladder) tubing to deliver intravenous infusions, measure arterial blood pressure, and collect urine, respectively. Rats were then gently placed in a Plexiglas holder and received an intravenous infusion of isotonic saline (20 µL/min) during a 2-h recovery period prior to experimentation for the stabilization of cardiovascular and renal excretory functions and to reach full consciousness. Mean arterial pressure (MAP) and heart rate (HR) were recorded continuously via the femoral artery cannula using BIOPAC data acquisition software (MP150 and AcqKnowledge 3.8.2; BIOPAC Systems) in conjunction with an external pressure transducer (P23XL; Viggo Spectramed) [18, 25–28].

#### Radiotelemetry probe implantation

Rats were anaesthetized with ketamine combined with xylazine (30 mg kg/ketamine, 3 mg kg/xylazine IP). A radiotelemetry probe (HD-S10, DSI, New Brighton, MN, USA) was implanted into the abdominal aorta via the left femoral artery and all animals recovered for 5–7 days prior to collection of baseline blood pressure data [6,9,10]. All animals were returned to their home cage after administration of penicillin (300,000 units/ml, 0.3 ml IM).

#### Afferent renal nerve ablation

In separate groups of 3- and 16-month-old male rats, bilateral selective afferent renal nerve ablation (ADNX) was performed 14 days prior to metabolic balance and acute blood pressure measurement. Selective ADNX was performed by direct application of 33 mM capsaicin to the renal nerves [12, 29]. Rats were anesthetized (sodium brevital, 20 mg/kg IP), and each kidney was exposed via a dorsal flank incision, and the renal artery and vein were gently separated from the surrounding fascia. Capsaicin (33 mM in isotonic saline containing 5% ethanol and 5% tween-20) was applied with a cotton swab, taking care to isolate the dissected renal artery and vein from the surrounding tissue to avoid off-target capsaicin exposure. Any excess capsaicin was dried before suturing the flank muscle and skin. In the sham denervation group, each kidney was exposed, and the renal artery and vein were visualized before suturing. The efficacy of selective ADNX was confirmed at the end of the study by (a) ELISA analysis of norepinephrine (NE) content in kidney tissue, to confirm no change in renal NE levels, according to the manufacturer’s instructions (IB89537, IBL America, MN) and (b) ELISA analysis of renal pelvic calcitonin gene related peptide (CGRP) content, to confirm significant reduction in renal pelvic CGRP levels, according to the manufacturer’s instructions (#589001, Cayman Chemicals, Ann Arbor, MI) [29]. All animals were returned to their home cage after administration of penicillin (300,000 units/ml, 0.3 ml IM).

#### Bilateral renal denervation

In separate groups of 3- and 16-month-old male rats, bilateral renal denervation (RDNX) or sham denervation was performed for 14 days prior to metabolic balance and acute blood pressure measurement. In these animals, standard techniques were used to remove the influence of both the afferent and efferent renal sympathetic nerve fibers [26, 30–32]. In brief, under sodium pentobarbital anesthesia (30 mg/kg IP), each kidney was exposed by a dorsal flank incision. Using a dissecting microscope, the renal vein and artery were dissected from the surrounding fascia and all visible renal nerve bundles were removed. After dissection, the renal artery was coated with a 10% phenol solution in ethanol to ensure the destruction of any remaining renal nerve fibers. In the sham denervation group, each kidney was exposed, and the renal artery and vein were visualized prior to suturing. The effectiveness of RDNX was confirmed at the end of the blood pressure measurement study by ELISA analysis of NE content in kidney tissue, to confirm an at least 90% reduction in renal NE content, as per the manufacturer’s instructions (ELISA IB89537, IBL America, MN) [32]. All animals were returned to their home cage after administration of penicillin (300,000 units/ml, 0.3 ml IM).

#### Subcutaneous osmotic minipump implantation

In certain studies, 3- and 16-month-old male Sprague–Dawley rats were anesthetized with sodium brevital (20 mg/kg IP). An osmotic minipump (2ML4, Alzet) was used to deliver a subcutaneous infusion of either DMSO/isotonic saline (50:50 solution, flow rate 5 μl/h) or hydrochlorothiazide (HCTZ; Sigma, St. Louis, MO; cat. no. H4759) dissolved in DMSO/isotonic saline (50:50 solution; HCTZ: 4 mg/kg^/^day; flow rate 5 μl/h) was surgically implanted subcutaneously in the subscapular region [18, 19, 25]. All animals were returned to their home cage after administration of penicillin (300,000 units/ml, 0.3 ml IM).

### Acute experimental protocols

#### Cardiovascular function

After a 2-h recovery period, baseline MAP was continuously recorded in naive conscious rats via the femoral artery cannula during a 1-h blood pressure measurement period or during the 1-h isotonic saline infusion period of the *Renal sodium transporter activity* protocol (see below). Reported MAP values represent the average MAP during the entire 1-h period [18, 19].

#### Assessment of vascular sympathetic tone

Following completion of the baseline blood pressure measurement, the peak change in MAP in response to an intravenous bolus of hexamethonium (30 mg/kg IV) was assessed. Baseline MAP was determined as the average MAP recorded over a 10-min control period prior to hexamethonium injection. After baseline MAP measurement, animals received an intravenous bolus of hexamethonium, and blood pressure was monitored for an additional 30-min period. The peak depressor response, assessed over a 60-s period, occurred within 5 min of injection [26, 30–33].

#### Metabolic balance studies

Metabolic studies were conducted in groups of 3-, 8-, and 16-month-old male and female Sprague–Dawley rats maintained on a 21-day NS or HS diet. In brief, rats were continuously housed individually in metabolic cages with ad libitum access to food and water. Following a 48-h acclimatization period, food and water consumption and urine output were measured. The 24-h sodium balance was calculated as the difference between dietary sodium intake and urinary sodium excretion. Urine samples were stored at − 80 °C until analysis.

#### Volume expansion

Male and female Sprague–Dawley rats aged 3, 8, and 16 months old, maintained on a lifelong NS diet, male rats aged 3 and 16 months old maintained on a 21-day high HS diet, male rats aged 16 months old receiving a 14-day s.c. HCTZ infusion (4 mg/kg/day) maintained on a lifelong NS diet, and male rats aged 16 months that underwent renal denervation 10–14 days previously maintained on a lifelong NS diet, underwent acute femoral vein, femoral artery, and bladder cannulation (*N* = 5–6/surgical group) (see “Surgical procedures”). Following a 2-h recovery period, conscious rats underwent an acute IV volume expansion (VE) protocol consisting of a 20-min control period (isotonic saline, 20 μL/min) followed by a 30-min VE period (isotonic saline, 5% body weight over 30 min) and a 90-min recovery period (isotonic saline, 20 μL/min) [25, 32, 34, 35]. MAP and HR were monitored continuously via the femoral artery cannula and BIOPAC. Urine was collected in consecutive 10-min increments. Immediately following the completion of the 120-min VE protocol, brain tissue was collected for PVN Fos immunohistochemistry as described below. In separate sets of Sprague–Dawley rats, tissue was collected for baseline PVN Fos immunohistochemistry immediately after the end of the 2-h surgical recovery period and a 20-min control period.

#### Ex vivo renal pelvis assay

Male and female Sprague–Dawley rats aged 3, 8, and 16 months old were maintained on a 21-day NS or HS diet (*N* = 6/sex/age) prior to conscious decapitation, plasma collection, and dissection of the renal pelvis from each kidney. Direct afferent renal nerve (ARN) responsiveness, assessed as substance P release, was tested in response to (1) a general ARN stimulus, NE (1250 pM; which acts on α_1_-adrenoceptors to drive an increase in ARN activity), and (2) a specific chemoreceptor stimulus (450 mM NaCl; the upper range of urinary sodium content) [12, 36]. Each renal pelvis was placed individually in a separate well of a 24-well plate containing 400 µL of HEPES medium and maintained at 37°C. Medium was replaced every 10 min during a 2-h equilibration period. During the experiment, medium was collected and replaced every 5 min during four control periods, one treatment period (1250 pM NE or 450 mM NaCl in HEPES), and four recovery periods. Each rat served as its own internal control, with one renal pelvis incubated with 1250 pM NE and one renal pelvis incubated with 450 mM NaCl during the treatment period. Medium was stored at − 80°C for analysis of substance P content via ELISA (Enzo Life Sciences, Farmingdale, NY, USA; cat#ADI-901–018). NE-evoked substance P release was calculated as the difference in substance P release between the treatment period and the average of the control periods [12].

#### Renal sodium transporter activity assay

Male and female Sprague–Dawley rats aged 3, 8, and 16 months old received an intravenous infusion for a 1-h control period (isotonic saline, 20 µL/min [18, 37]), a 1-h epithelial sodium channel (ENaC) blockade period (amiloride, ENaC antagonist; 2 mg/kg bolus followed by 2 mg/kg/h at 20 µL/min [18, 19, 25, 37]) and a 1-h sodium chloride cotransporter (NCC) blockade period during which ENaC blockade were maintained (hydrochlorothiazide (HCTZ), NCC antagonist; 2 mg/kg bolus followed by 2 mg/kg/h HCTZ + 2 mg/kg/h amiloride at 20 µL/min [18, 19, 25, 37]). The use of amiloride allows for the isolation of the NCC’s contribution to urinary sodium excretion. MAP measurements were obtained during the 1-h control period. Throughout the protocol, urine was collected via the bladder cannula in 10-min increments to assess urinary sodium concentration. Estimated NCC activity was assessed as the peak natriuretic response (∆UNaV; µeq/min) to HCTZ, calculated by subtracting the average UNaV from the last two 10-min periods of ENaC blockade from the maximum UNaV during NCC blockade (occurred within the first two 10-min periods of NCC blockade in all animals). Estimated ENaC activity was assessed as the peak natriuretic response (∆UNaV; µeq/min) to amiloride, calculated by subtracting the average UNaV from the last two 10-min control periods from the maximum UNaV during ENaC blockade (occurred within the first two 10-min periods of ENaC blockade in all animals). Following protocol completion, rats were decapitated while conscious, and both kidneys were collected and immediately frozen at − 80 °C for measurement of the NCC and associated regulatory kinases.

#### Assessment of estimated glomerular filtration rate

Estimated glomerular filtration rate (GFR) was assessed using a transdermal GFR monitor (Medibeacon GmbH, Mannheim, Germany) in randomly selected male rats that subsequently underwent an acute VE or an acute renal sodium transporter assay [38]. Following acute femoral vein, artery, and bladder cannulation, sodium methohexital anesthesia was briefly maintained while the monitor was secured to a depilated region on the back of the rat. In these rats, an intravenous bolus of FITC-sinistrin (5 mg per 100 g body weight) was administered 30 min after the start of the surgical recovery period, and the monitor was recovered at the end of the acute experimental protocol. As such, as demonstrated in Figure S6, estimated GFR was calculated from elimination kinetics prior to the start of an acute VE or renal sodium transporter assay. The elimination kinetics of FITC-sinistrin were analyzed using proprietary software supplied with the transdermal monitor and GFR was calculated as previously published [38].

#### Radiotelemetry studies

A separate group of male Sprague–Dawley rats underwent radiotelemetry probe implantation (see “Surgical procedures”) and blood pressure data were recorded by radiotelemetry (Dataquest A.R.T. 4.2 software (DSI)) via scheduled sampling for 10 s every 10 min. Rats were maintained on a normal salt (0.6% NaCl) diet across the lifespan. Twenty-four-hour averages of MAP were determined.

#### PVN Fos immunohistochemistry

Rats that underwent a 120-min acute VE study or a control 2-h recovery period were deeply anesthetized with sodium methohexital (10 mg/kg IV) and underwent transcardiac perfusion with 0.1 M phosphate-buffered saline (PBS) followed by 4% paraformaldehyde (PFA). Brains were removed and submerged in 4% PFA overnight followed by 30% sucrose for 2 days. The PVN was sectioned into three sets of serial 40-µm coronal sections, which were stored free-floating in cryoprotectant at − 20°C until Fos immunohistochemistry was performed [12, 39] on samples selected at three rostral-caudal levels from bregma—level 1 (− 1.60mm), level 2 (− 1.88mm), and level 3 (− 2.12mm).

In brief, Fos immunohistochemistry was performed on a set of free-floating PVN sections from each rat as previously described [39, 40]. Sections stored in cryoprotectant were brought to room temperature and were rinsed twice for 30 min in 0.1 M PBS. Sections were incubated in 0.3% hydrogen peroxide in dH_2_0 for 30 min and rinsed in 0.1 M PBS for 30 min. Sections were blocked in PBS diluent (0.1 M PBS containing 3% normal horse serum and 0.25% Triton X-100) for 2 h and then incubated with primary antibody (anti-Fos Ab-5, Calbiochem, San Diego, CA; 1: 30,000 in PBS diluent) for 48 h at 4°C. Sections were rinsed twice in 0.1 M PBS for 30 min and then incubated with secondary antibody (biotinylated horse anti-rabbit IgG, Vector Laboratories, Burlingame, CA, USA; 1:200 in PBS diluent) for 2 h at room temperature. Sections were then incubated with an avidin-peroxidase conjugate (ABC-Vectastain Kit; Vector Laboratories) and developed using 0.04% 3,3′-diaminobenzidine hydrochloride and 0.04% nickel ammonium sulfate in 0.1 M PBS. Sections were mounted on gelatin-coated slides and dehydrated using a graded series of alcohols followed by xylenes. Coverslips were placed on the slides using Permount mounting medium. Tissue sections were imaged using an Olympus microscope (BX41) and an Olympus DP70 digital camera with DP MANAGER software (v 2.2.1) (Olympus, Center Valley, PA, USA). The PVN was sampled at three rostral–caudal levels, and two sets of tissue from each animal were analyzed. The Fos‐positive cell counts were quantified by participants blinded to the experimental conditions using National Institutes of Health Image J software (NIH, Bethesda, MD, USA), and the counts for each PVN subnucleus were averaged for each animal.

### Renal CGRP immunohistochemistry

Formalin-preserved kidneys underwent CGRP immunohistochemistry as previously described [29]. In brief, paraffin-embedded kidneys were cut transversely at 5 μm and mounted on glass slides. Sections were deparaffinized and subjected to heat-induced epitope retrieval in a Decloaking Chamber (model NxGen DC2012; Biocare Medical, Concord, CA) using Antigen Decloaker solution (CB910M; Biocare Medical) at 95 °C. Endogenous peroxidases were then blocked with Peroxidazed 1 (PX968M; Biocare Medical). Sections were incubated in Protein Block Serum-Free (Dako, Glostrup, Denmark; X0909) at room temperature and then in a polyclonal CGRP antibody (BML-CA1134-0100; Enzo Life Science, Farmingdale, NY) diluted to 1:500 in DaVinci Green Universal Diluent (PD900; Biocare Medical) at 4 °C overnight. Sections were incubated in an anti-rabbit secondary antibody conjugated to HRP (cat. 31,460; Invitrogen, Rockford, IL) diluted to 1:1000 at room temperature for 1 h. Betazoid DAB Chromogen (BDB2004MM; Biocare Medical) was then applied to each slide for 2 min at room temperature. The sections were then counterstained with Maye’s hematoxylin undiluted (26,043–06; Electron Microscopy Sciences, Hatfield, PA) for 1–2 min. Each slide was dehydrated in graded ethanol and xylene, and a coverslip was mounted with Permount Toluene Solution mounting medium (UN1294 toluene solution; Fisher Chemical, Waltham, MA). Tissue sections were imaged using a Keyence BZ-9000 microscope set to brightfield, and CGRP levels were expressed as GCRP positive area in μM^2^ per μM pelvic wall.

### Renal pelvic CGRP content

Renal pelvic CGRP content was assayed as previously described. In brief, kidneys were immediately removed, and the renal pelvis was carefully dissected and frozen at − 80 °C until assayed for CGRP content using a commercially available ELISA kit (Cayman Chemicals, Ann Arbor, MI; item number 589001) as per the manufacturer’s instructions. To eliminate any interassay variance, all pelvic samples were run on single 96-well ELISA plates.

### Kidney protein extract preparation

Kidneys harvested from rats after decapitation and following completion of the acute experiments were immediately stored at − 80°C. Approximately 200 mg of kidney cortex tissue was homogenized on ice using a Potter Elvehjem tissue grinder (Kimble, Cat. No. 885510–0021) in a homogenizing buffer containing Halt protease inhibitors cocktail (Thermoscientific, Cat. No. 78429) and PhosSTOP phosphatase inhibitor (Roche, Cat. No. 04906845001) and then centrifuged at 4000* g* for 15 min at 4 °C [19, 25, 41]. The supernatant was collected and centrifuged again at 17,000* g* for 1 h at 4 °C. The pellet was then resuspended in cell lysis buffer (9803S Cell Signaling Technology, MA) [19, 25, 41]. A BCA assay was used to determine the total protein content. Prepared protein extracts were stored at − 80°C prior to use in immunoblotting studies.

### Immunoblotting

Kidney cortex protein extracts were loaded at 20 µg of total protein per lane with precision plus protein kaleidoscope standard included (BIO-RAD, Cat. No. 1610375). Nitrocellulose membranes (GE, Cat. No. 10600096) were blocked in 5% blocking grade blocker (BIO-RAD, Cat. No. 170–6404) for 30 min and probed overnight at 4°C with primary antibodies in 0.1% TBS-Tween. Membranes were washed with 0.1% TBS-Tween and incubated for 2 h with secondary antibodies in 0.1% PBS-Tween at room temperature. Membranes were visualized using chemiluminescence (SuperSignal West Pico Plus, Cat. No. 34580; Thermo Scientific). Semi-quantitative analysis was performed using Image J software (NIH, Bethesda, MD, USA), and protein expression was normalized to total protein using Coomassie staining (Bio-Safe, Cat. No. 1610786, BIO-RAD) [19]. A series of control validation gels were run using control protein standards (Figures S18-S20)—these gels which validate the specificity of the antibodies are publicly available at https://figshare.com/articles/journal_contribution/AJP_Renal_online_supplement/23261495. To address antibody specificity and cross-reactivity protein standards for SPAK, OxSR1 and WNK4 were loaded on the same gel at a 0.5 and 1 μg concentration. The SPAK control was SPAK human recombinant protein that contains full length human protein (96% homology with *Rattus norvegicus*) that has an N-terminal His Tag with a predicted molecular weight of approximately 63 kDa. The OxSR1 control was OxSR1 human recombinant protein containing amino acids 1–527 of the human OxSR1 protein (74% homology with *Rattus norvegicus*) that has an N-terminal His Tag and a predicted molecular weight of approximately 60 kDa. The WNK4 control was WNK4 human recombinant protein containing amino acids 1–444 of the human WNK4 (90.32% homology with *Rattus norvegicus*) that has a GST tag and a predicted molecular weight of approximately 77 kDa. The NCC antibodies (total and phospho) used in this study were developed by Dr. Fenton’s laboratory and have been extensively validated by multiple groups and have been previously used by our laboratory generating results consistent with the current studies [18, 19, 25]. The WNK1 antibody used in these studies has been previously validated with positive and negative controls [42] and has been previously published by our laboratory in rat kidney tissue [19, 25]. A phosphatase (Sigma, P9614), which removes phosphate groups from serine, threonine, tyrosine, and histidine residues, was added to kidney lysate in a 1:20 ratio and incubated at 30℃ for 30 min; 20 µg of protein and untreated and phosphatase-treated samples were run next to each other. As shown in Figure S21, this significantly downregulated the signal detected by pNCC53. Antibodies and dilutions and standards and concentrations used are provided in Supplemental Table 1.

### Assessment of glomerulosclerosis and mesangial expansion

Kidneys were bisected and immersed in neutral buffered formalin. The tissue was stored in formalin at room temperature until processing and embedding. Periodic acid-Schiff staining with a hematoxylin counterstain was performed by the Boston University Chobanian & Avedisian School of Medicine Experimental Pathology Laboratory Service Core. Approximately 40 glomeruli per animal were imaged at 60 × magnification using an Olympus microscope (BX41) and an Olympus DP70 digital camera with DP MANAGER software (v 2.2.1) (Olympus, PA, USA). Glomerulosclerosis and mesangial expansion were evaluated and scored separately by two blinded reviewers [43]. The scores noted by each reviewer were averaged. Glomerulosclerosis was defined as the obliteration of capillary lumens, folding, and thickening of the glomerular basement membrane, and loss of cellular elements from the glomerular tuft, while mesangial expansion was defined as the presence of periodic acid-Schiff-positive material in the mesangium. Semi-quantitative scores were obtained using a previously established scale in which the percentage of each individual glomerulus involved was denoted as 0 (no lesion) or 1, 2, 3, or 4, corresponding to 25%, 50%, 75%, and 100% glomerular area.

### Analytical techniques

Urine volume was determined gravimetrically, assuming 1 g = 1 mL. Urine sodium and potassium concentrations were determined by flame photometry (model 943; Instrumentation Laboratories), and urinary aldosterone (ALDO; ADI-900–173, Enzo Life Science), plasma NE (IB89552, Immuno-Biological Labs America), renal NE (IB89537, Immuno-Biological Labs America), urinary angiotensinogen (UAGT; UAGT IBL #27414), plasma estradiol (17β-estradiol) (IB79329 Immuno-Biological Labs America), and urinary kidney injury molecule-1 (KIM-1; ab119597, Abcam) levels were determined using ELISA as per the manufacturers’ instructions. Fractional excretion of sodium was determined using standard techniques as previously described by our laboratory [32].

### Statistical analysis

All data are expressed as mean ± SD. The magnitude of change in cardiovascular and renal excretory parameters at different time points was compared with the mean group control value by a one-way repeated-measures analysis of variance (ANOVA) with subsequent Dunnett’s test. Differences between treatment groups were assessed by a two-way repeated measure ANOVA, with treatment group as one fixed effect and time as the other, including interaction. The time (min) was used as a repeated factor. Post hoc analysis was performed using Bonferroni test to compare variations between groups. Comparisons were made between NS and HS intake groups using a two-tailed Student’s *t*-test. Statistical analysis was carried out using Prism 9 (GraphPad Software, CA).

## Results

### Impact of age on BP and sympathetic outflow

**Fig. 1:**
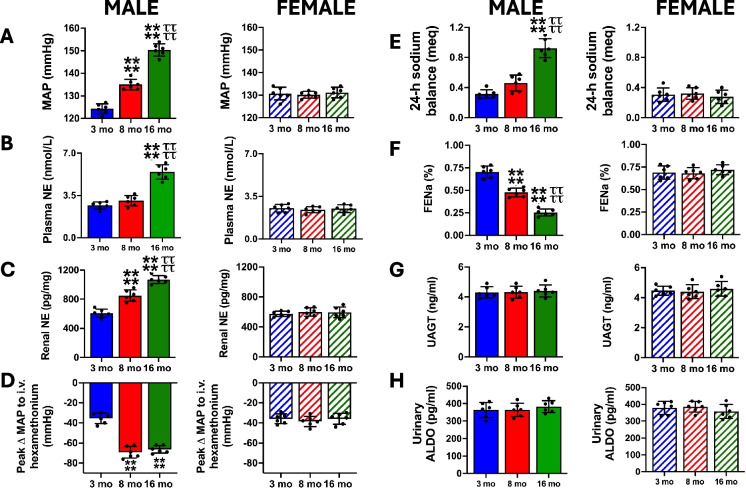
Impact of aging on blood pressure, indices of sympathetic tone, renal sodium handling, and the renin–angiotensin–aldosterone system. **A** Mean arterial pressure (MAP; mmHg), **B** circulating plasma NE content (nmol/L), **C** renal NE content (pg/mg), **D** peak ΔMAP (mmHg) in response to i.v. hexamethonium (30 mg/kg), **E** 24-h sodium balance (meq), **F** FENa (%), **G** UAGT (ng/ml), and **H** urinary ALDO (pg/ml) in 3-, 8-, and 16-month-old male and female Sprague–Dawley rats maintained on a lifelong normal salt intake (NS; 0.6% NaCl). *N* = 6 per group, mean ± SD. MAP, mean arterial pressure; NE, norepinephrine; FENa, fractional excretion of sodium; UAGT, urinary angiotensinogen; ALDO, aldosterone. **P < 0.01 vs. respective within sex 3-month-old group value. ****P < 0.0001 vs. respective within 3-month-old group value. ^ττττ^*P* < 0.0001 vs. respective within sex 8-month-old group value

In male Sprague–Dawley rats, we observed a progressive increase in BP that correlated with increasing age (Pearson’s *R*^2^ 0.874, *P* < 0.05; Fig. 1A). In the same conscious rats, we observed an increase in the vascular response to ganglionic blockade, indicative of increased sympathetic tone to the vasculature, which was of a similar magnitude at both 8 and 16 months of age (Fig. 1D) despite the increase in BP between 8 and 16 months of age. Additionally, we detected a progressive age-dependent increase in global and renal sympathetic tone at 8 and 16 months of age (Fig. 1B, C). In the same animals in which BP and indices of sympathetic tone were measured, renal sodium retention, assessed as 24-h sodium balance and fractional excretion of sodium (FENa), also progressively increased with age (Fig. 1E, F). In contrast to the effect of age on sympathetic outflow, there was no detectable effect of increased age on markers of systemic and intrarenal renin–angiotensin–aldosterone system (RAAS) activity (Fig. 1G, H). Validating the increase in BP with age in male rats observed in acutely instrumented rats in a sub-group of radiotelemetered male rats, we observed an age-dependent increase in BP of the same magnitude and time course as seen in acutely instrumented rats (Figure S1). Demonstrating a clear sex difference in the effect of age on BP, female Sprague–Dawley rats that exhibit no alteration in uterus weight or change in plasma estradiol levels at 16 months of age (Figure S2), does not develop an age-dependent increase in BP, renal sodium retention, indices of sympathetic outflow, or detectable alterations in the RAAS (Fig. 1A–H).

### Impact of age on the renal excretory and PVN neural responses to acute volume expansion

**Fig. 2:**
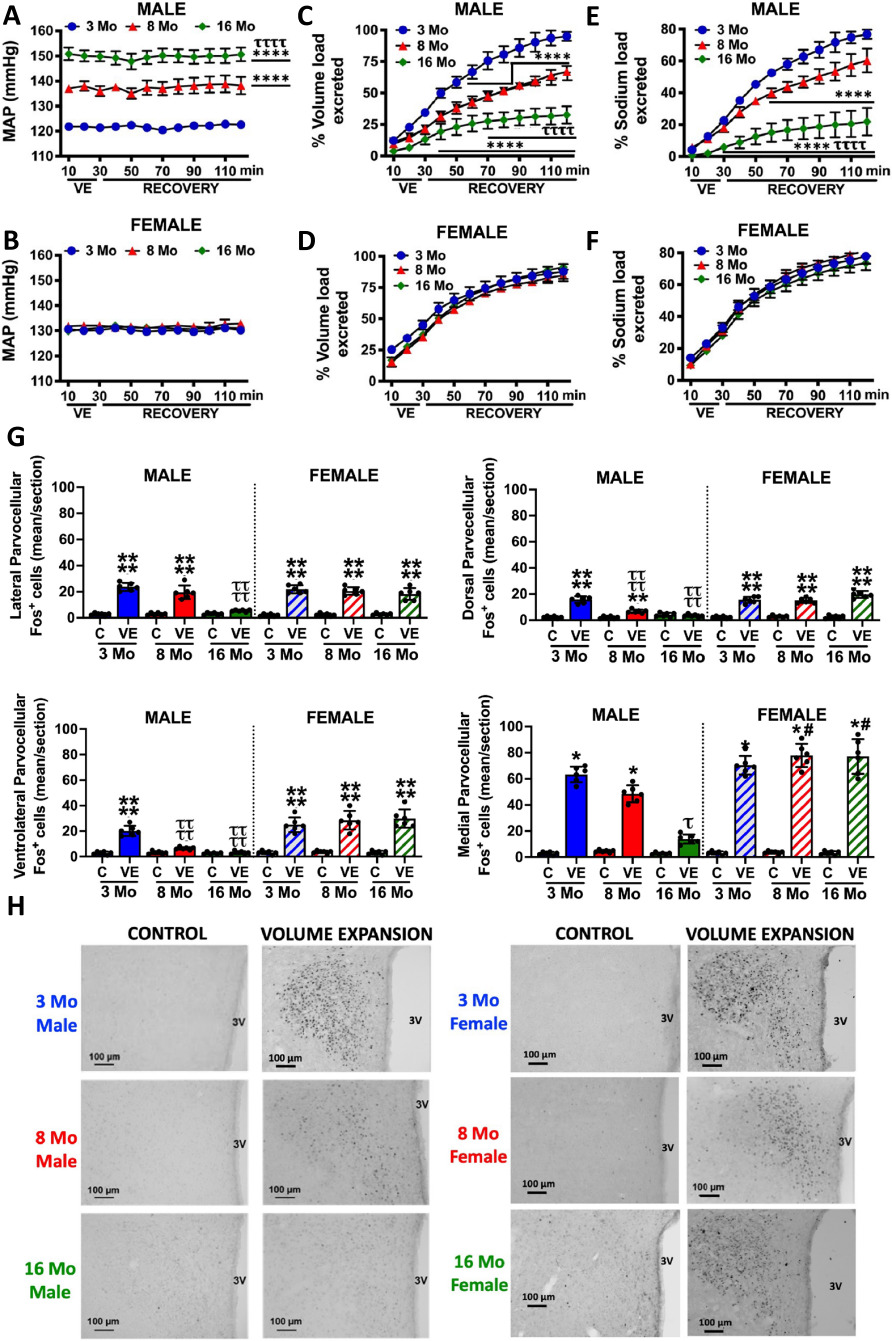
Impact of aging on the blood pressure, renal excretory and PVN neuronal responses to an acute volume expansion. **A**, **B** Mean arterial pressure (MAP; mmHg), **C**, **D** urinary volume (% volume load excreted), and **E**, **F** urinary sodium excretion (% sodium load excreted) in response to a 30-min isotonic saline volume expansion (VE) of 5% body weight followed by a 90-min recovery period in conscious 3-, 8-, and 16-month-old male and female Sprague–Dawley rats maintained on a lifelong normal salt intake (NS; 0.6% NaCl), **G** neuronal activation (c‐fos‐positive cell count) in the lateral parvocellular, ventrolateral parvocellular, medial parvocellular, and dorsal parvocellular regions of the paraventricular nucleus (PVN) of the hypothalamus sampled at three rostral-caudal levels from bregma—level 1 (− 1.60mm), level 2 (− 1.88mm), and level 3 (− 2.12mm) following VE or a 2-h surgical recovery and control period (baseline group) in conscious 3-, 8-, and 16-month-old male and female Sprague–Dawley rats maintained on a lifelong normal salt intake (NS; 0.6% NaCl), and **H** representative images from level 2 of the PVN. *N* = 6 per group mean ± SD. MAP, mean arterial pressure. *P < 0.05 vs. respective group control value, denoted **C**; ****P < 0.0001 vs. respective group control value or respective 3-month-old group value, ^τ^*P* < 0.05 vs. respective within sex 3-month-old VE group value, ^ττττ^*P* < 0.05 vs. respective within sex 3-month-old VE group value or respective 8-month-old group value

The administration of an acute i.v. 5% body weight VE over a 30-min period, which increases renal pelvic pressure to activate mechanosensitive ARN [12], did not alter BP irrespective of sex or age (Fig. 2A, B). In response to an acute VE, normotensive 3-month-old male rats exhibited robust natriuretic and diuretic responses, excreting approximately 80% and 95% of the administered sodium and fluid load, respectively (Fig. 2C–F). In aged male rats, with elevated BP, we observed a progressive age-dependent attenuation of the natriuretic and diuretic responses to an acute VE, resulting in the excretion of approximately 21% of the sodium and 32% of the administered volume load at 16 months of age. Age had no impact on baseline sodium or potassium excretion or urine output during the control period (Supplemental Table 2). With respect to the PVN neural responses, in the same animals for which the physiology is presented in Fig. 2A–C, an acute VE resulted in increased Fos staining in PVN parvocellular subnuclei in 3-month-old male rats (Fig. 2G, H); however, the most significant changes were observed in the medial parvocellular region. Significantly, Fos induction in sympathetic parvocellular subnuclei was progressively and significantly attenuated in aged male rats with no impact of age on VE-evoked activation of magnocellular neurons (Fig. 2G, H). In contrast, the profound natriuretic, diuretic, and PVN neuronal responses to an acute VE were not affected by age in female rats (Fig. 2). The borders of the identified PVN sub-regions are defined in a representative annotated image (Figure S3).

### Impact of age on ARN activity

**Fig. 3:**
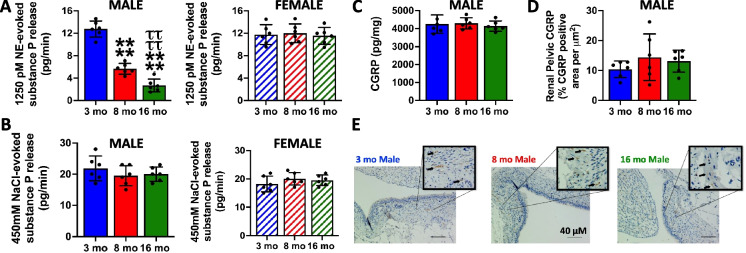
Impact of aging on the role of the renal nerves in renal sodium balance and BP regulation. Ex vivo renal pelvis substance P release (pg/min) in response to **A** 1250 pM norepinephrine (NE) and **B** 450 mM NaCl in 3-, 8-, and 16-month-old male and female Sprague–Dawley rats maintained on a lifelong normal salt intake (NS; 0.6% NaCl), **C** renal pelvis CGRP content (pg/mg), **D** renal pelvic CGRP expression (% CGRP positive area per μm^2^), **E** representative immunohistochemistry (IHC) images stained for CGRP in 3-, 8-, and 16-month-old male Sprague–Dawley rats maintained on a lifelong normal salt intake (NS; 0.6% NaCl). *N* = 6 per group, mean ± SD. NE, norepinephrine. ****P < 0.0001 vs. respective within 3-month-old group value. ^ττττ^*P* < 0.0001 vs. respective within sex 8-month-old group value

Treatment of an ex vivo renal pelvic preparation with norepinephrine (NE), a general ARN stimulus that activates both mechano- and chemosensitive ARN terminals, evoked an equivalent increase in the release of substance P in 3-month-old male and female rats. The response of the ARN to stimulation with NE was significantly and progressively attenuated in 8-month and 16-month-old male, but not female, rats when compared with the response observed in 3-month-old rats (Fig. 3A). In contrast, ARN activity in response to direct stimulation with 450 mM NaCl, a selective chemoreceptor stimulus, was not altered by age in either sex (Fig. 3B). To assess if the observed decrease in the ARN activity with age in male rats reflects alterations in the presence of the ARN, we assayed CGRP levels and expression. These data show that increased age does not affect renal pelvic GCRP levels or the renal expression of CGRP (a marker for the presence of the ARN) in male rats (Fig. 3C–E).

### Impact of age on the salt sensitivity of BP

**Fig. 4:**
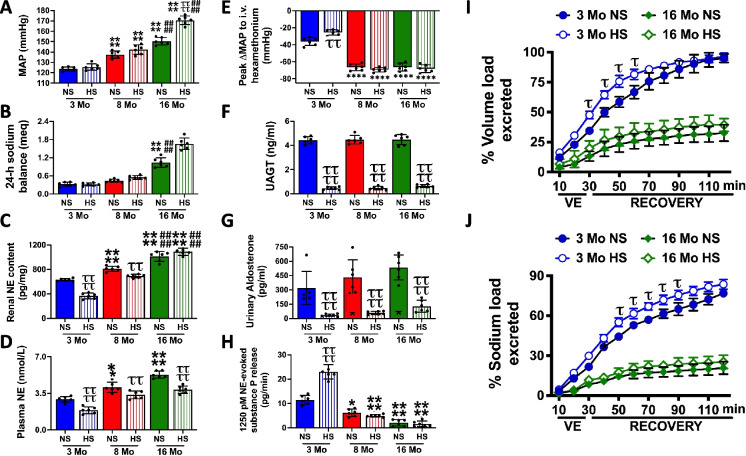
Impact of aging on the salt sensitivity of BP, indices of sympathetic tone, renal sodium handling, and the renin–angiotensin–aldosterone system. **A** Mean arterial pressure (MAP; mmHg), **B** 24-h sodium balance (meq), **C** renal NE content (pg/mg), **D** circulating plasma NE content (nmol/L), **E** peak ΔMAP (mmHg) in response to i.v. hexamethonium (30 mg/kg), **F** UAGT (ng/ml), **G** urinary aldosterone content (pg/ml), **H** ex vivo renal pelvis substance P release (pg/min) in response to 1250 pM norepinephrine (NE), in 3-, 8-, and 16-month-old male Sprague–Dawley rats maintained on a lifelong normal salt intake (NS; 0.6% NaCl) and challenged for 21 days with a NS or high salt intake (HS; 4% NaCl) and **I** urinary volume (% volume load excreted) and **J** urinary sodium excretion (% sodium load excreted) in response to a 30-min isotonic saline volume expansion (VE) of 5% body weight followed by a 90-min recovery period in conscious 3- and 16-month-old male Sprague–Dawley rats maintained on a lifelong normal salt intake (NS; 0.6% NaCl) and challenged for 21 days with a NS or high salt intake (HS; 4% NaCl), *N* = 6 per group, mean ± SD. MAP, mean arterial pressure; NE, norepinephrine; UAGT, urinary angiotensinogen. *P < 0.05 vs. respective within diet 3-month-old group, ****P < 0.0001 vs. within diet 3-month-old group, ^τ^*P* < 0.05 vs. respective within age NS group, ^ττ^*P* < 0.01 vs. respective within age NS group, ^ττττ^*P* < 0.0001 vs. respective within age NS group. ^####^*P* < 0.0001 vs. respective within diet 8-month-old group

Three-month-old male and female rats exhibited a salt-resistant phenotype when challenged with a 21-day HS intake (an established experimental paradigm used to assess the salt sensitivity of BP in rats), with no detectable change in BP or 24-h sodium balance (Fig. 4 and Figure S4). The phenotype of salt resistance was maintained in 8-month-old male and female rats (Figure S4). However, in 16-month-old male rats, a 21-day HS diet evoked the salt sensitivity of BP and renal sodium retention (Fig. 4A, B). Thus, we elected to investigate the mechanism underlying the phenotypic switch from salt resistance to the salt sensitivity of BP in male Sprague–Dawley rats. In 3-month-old male rats, salt resistance was accompanied by dietary sodium-evoked suppression of global, vascular, and renal sympathetic tone; suppression of indices of the RAAS activity; and increased ARN activity (assessed as NE-evoked SP release) (Fig. 4C–H). However, while 8-month-old male rats exhibited salt resistance and suppression of the RAAS as observed in 3-month-old rats, dietary sodium-evoked suppression of vascular sympathetic tone was abolished, dietary sodium-evoked suppression of global and renal sympathetic tone was attenuated, and no dietary sodium-evoked increase in ARN activity was observed (Fig. 4C–H). In 16-month-old male rats, hypertension was significantly exacerbated with a 21-day HS diet, by approximately 20 mmHg, indicating the development of the salt sensitivity of BP (Fig. 4A). The salt sensitivity of BP was accompanied by impaired dietary sodium-evoked suppression of global sympathetic tone, a failure to suppress renal or vascular tone and markedly reduced ARN release of substance P (Fig. 4C–H). Further, while an acute VE following HS intake did not alter BP in young or aged rats (Figure S5), there was no enhanced natriuretic and diuretic response to an acute VE following HS intake in aged male rats—in contrast to the sensitized responses observed in young male rats (Fig. 4I, J). Despite the marked age-related changes in BP and renal sodium handling, we observed no changes in renal hemodynamics or structure in aged male rats. There was no detectable effect of age or dietary salt intake on estimated GFR, proteinuria, urinary kidney injury molecule-1 levels, glomerulosclerosis, or mesangial expansion (Figures S6-S8).

### Impact of age and high dietary sodium intake on NCC expression and activity

**Fig. 5:**
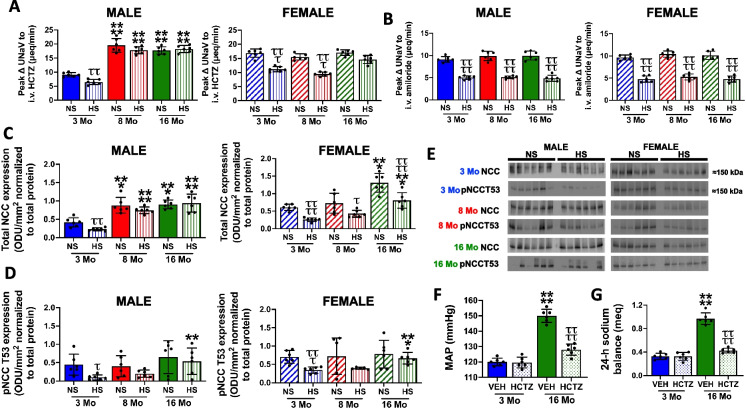
Impact of age and high dietary sodium intake on NCC expression and activity. **A** In vivo NCC activity expressed as peak natriuretic response (ΔUNaV) to intravenous hydrochlorothiazide (HCTZ; 2 mg/kg bolus, 2 mg/kg hour infusion), **B** in vivo ENaC activity expressed as peak ΔUNaV to intravenous amiloride (2 mg/kg bolus, 2 mg/kg hour infusion), **C** total NCC expression (optical density units (ODU)/mm^2^ normalized to total protein), **D** pNCCT53 expression (ODU/mm^2^ normalized to total protein), and **E** immunoblots for total NCC and pNCCT53 in 3-, 8-, and 16-month-old male and female Sprague–Dawley rats maintained on a lifelong normal salt intake (NS; 0.6% NaCl) and challenged for 21 days with a NS or high salt intake (HS; 4% NaCl), and **F** mean arterial pressure (MAP; mmHg), and **G** 24-h sodium balance (meq) in 3-month-old and 16-month-old male Sprague–Dawley rats maintained on a lifelong normal salt intake (NS; 0.6% NaCl) that received a 21-day s.c. osmotic minipump infusion (flow rate 5 μl/h) of vehicle (50:50 DMSO:Saline or HCTZ (4 mg/kg/day). *N* = 6 per group, mean ± SD. MAP, mean arterial pressure; NCC, sodium chloride cotransporter; ENaC, epithelial sodium channel; HCTZ, hydrochlorothiazide; UNaV, urinary sodium excretion. ***P < 0.001 vs. respective within diet 3-month-old group, **P < 0.01 vs. respective withing diet 3-month-old group, ****P < 0.0001 vs. respective within diet 3-month-old group or VEH treatment group. ^τ^*P* < 0.05 vs. respective within age NS group, ^ττ^*P* < 0.01 vs. respective within age NS group, ^τττ^*P* < 0.001 vs. respective within age NS group, ^ττττ^*P* < 0.0001 vs. respective within age NS group or VEH treatment group

Male rats exhibit an age-dependent increase in in vivo estimated NCC activity, assessed as the peak natriuretic response to hydrochlorothiazide (HCTZ) and total NCC protein expression (Fig. 5A, C, D), which was accompanied by increased plasma NE and renal NE content (Fig. 1). However, we observed no age-dependent increase in NCC Thr53 phosphorylation (pNCC) or estimated epithelial sodium channel (ENaC) activity (expressed as the peak natriuretic response to amiloride) (Fig. 5B, D, E). Significantly, in 16-month-old male rats, NCC antagonism for 14 days with an s.c. infusion of HCTZ abolished age-dependent hypertension and restored 24-h sodium balance without altering the renal excretory responses to an acute 5% isotonic saline VE (Figs. 5F, G, S9).

In response to 21-day HS intake, young male and female rats exhibit dietary sodium-evoked suppression of in vivo estimated NCC activity, in vivo estimated ENaC activity, and sympathetic outflow (Figs. 1, 5A, B) without observable dietary sodium-evoked alterations in baseline sodium or potassium excretion during the renal transporter assay (Supplemental Table 3). Reduced in vivo NCC activity in young rats was paralleled by dietary sodium-evoked suppression of both total NCC expression and NCC Thr53 phosphorylation, without altering the phosphorylated to total NCC ratio, normalized to Coomassie blue (Fig. 5C, D, E, Figure S10, S11). Critically, aged male rats fed a HS diet fail to suppress total NCC levels, estimated in vivo NCC activity, total NCC expression levels, or Thr53 phosphorylation (Fig. 5).

In contrast, in female rats, we observed no age-dependent increase in NCC activity, assessed as estimated in vivo activity and NCC phosphorylation, despite an increase in total NCC protein expression at 16 months (Fig. 5A–C). Additionally, we observed dietary sodium-evoked suppression of total NCC expression in female rats at all ages (Fig. 5C, D, Figure S12). In 3- and 8-month-old female rats, we observed dietary sodium-evoked suppression of in vivo estimated NCC activity that was absent in 16-month-old females (Fig. 5A).

### Impact of age on NCC regulatory kinases

**Fig. 6:**
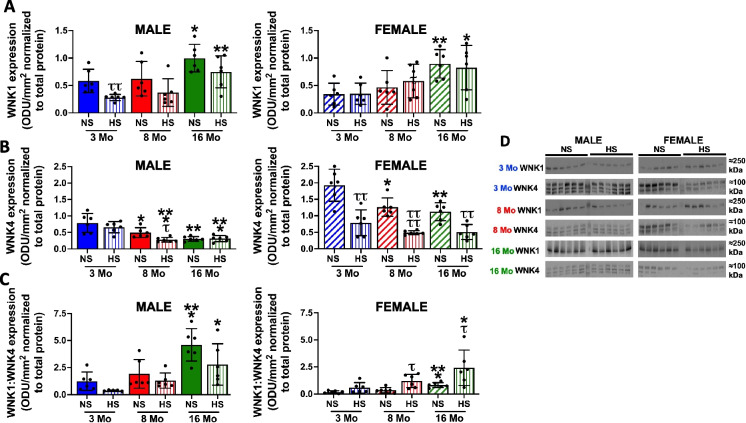
Impact of age and high dietary sodium intake on WNK regulation. **A** Total WNK1 expression (optical density units (ODU)/mm^2^ normalized to total protein), **B** total WNK4 expression (ODU/mm^2^ normalized to total protein), **C** WNK1:WNK4 expression ratio, and **D** corresponding immunoblots, in 3-, 8-, and 16-month-old male and female Sprague–Dawley rats maintained on a lifelong normal salt intake (NS; 0.6% NaCl) and challenged for 21 days with a NS or high salt intake (HS; 4% NaCl). WNK, with-no-lysine kinase. *N* = 6 per group, mean ± SD. *P < 0.05 vs. respective within diet 3-month-old group, **P < 0.01 vs. respective within diet 3-month-old group, ***P < 0.001 vs. respective within diet 3-month-old group, ^τ^*P* < 0.05 vs. respective within age NS group, ^ττ^*P* < 0.01 vs. respective within age NS group, ^ττττ^*P* < 0.0001 vs. respective within age NS group

**Fig. 7:**
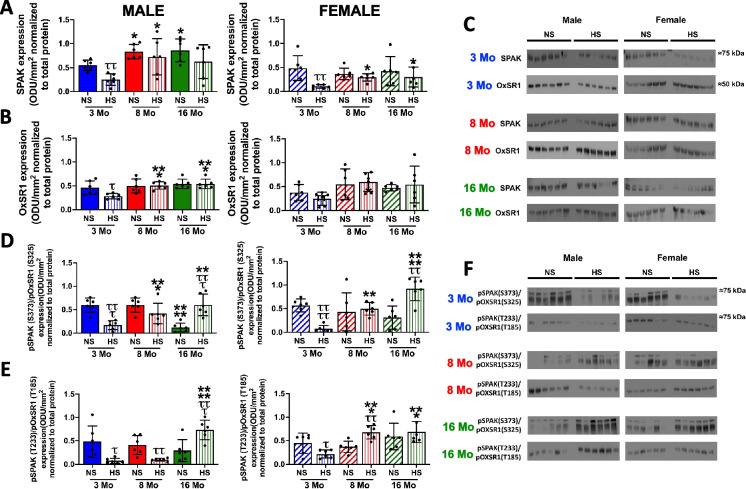
Impact of age and high dietary sodium intake on SPAK and OxSR1 regulation. **A** Total SPAK expression (optical density units (ODU)/mm^2^ normalized to total protein), **B** total OxSR1 expression (ODU)/mm^2^ normalized to total protein), **C** corresponding SPAK and OxSR1 immunoblots, **D** pSPAK (S373)/pOxSR1 (S325) expression (ODU)/mm^2^ normalized to total protein), **E** pSPAK (T233)/pOxSR1 (T185) expression (ODU)/mm^2^ normalized to total protein), and **F** corresponding pSPAK/pOxSR1 immunoblots in 3-, 8-, and 16-month-old male and female Sprague–Dawley rats maintained on a lifelong normal salt intake (NS; 0.6% NaCl) and challenged for 21 days with a NS or high salt intake (HS; 4% NaCl). *N* = 6 per group, mean ± SD. *P < 0.05 vs. respective within diet 3-month-old group, **P < 0.01 vs. respective within diet 3-month-old group, ***P < 0.001 vs. respective within diet 3-month-old group, ****P < 0.0001 vs. respective within diet 3-month-old group, ^ττ^*P* < 0.01 vs. respective within age NS group, ^τττ^*P* < 0.001 vs. respective within age NS group, ^ττττ^*P* < 0.0001 vs. respective within age NS group

In male and female rats, we observed an increase in WNK1 expression at 16 months, with a greater magnitude of WNK1 increase in female vs. male rats (Fig. 6A). In relation to WNK4 expression, both male and female rats exhibit reduced WNK4 levels at 8 and 16 months. These changes result in a greater WNK1:WNK4 ratio with age in both sexes. In both male and female rats, we observed no age-dependent change in OxSR1 expression levels and an increase in SPAK expression in aged male rats (Fig. 7A, B). Regarding SPAK and OxSR1 activity, assessed as protein phosphorylation, there was no age-dependent change in pSPAK(T233)/pOxSR1(T185); however, there was a sex-specific decrease in pSPAK(S373)/pOxSR1(S325) in aged male but not female rats (Fig. 7D, E).

### Impact of age and high dietary sodium intake on NCC regulatory kinases

High dietary salt intake for 21 days evoked sex-dependent differential WNK kinase regulation. In response to a HS diet in 3-month-old rats, we observed a selective dietary sodium-evoked suppression of WNK1 expression with no change in WNK4 levels. In contrast, in female rats, we observed selective suppression of WNK4 with no change in WNK1 levels (Fig. 6). Total SPAK expression and SPAK/OxSR1 phosphorylation were reduced in response to HS diet in male and female 3-month-old rats, and OxSR1 was downregulated only in young male rats (Fig. 7).

In aged male rats on a HS diet, we observed a loss of dietary sodium evoked suppression of WNK1, SPAK, and OxSR1 at 8 and 16 months (Figs. 6 and 7). Dietary sodium intake resulted in increased serine SPAK/OxSR1 phosphorylation at 8 and 16 months and threonine phosphorylation at 16 months (Fig. 7). In aged female rats, high dietary sodium intake did not affect the age-related increase in WNK1 and a dietary sodium-evoked suppression of WNK4 was maintained at all ages. This resulted in a divergent sex-dependent HS-evoked shift in the WNK1:WNK4 ratio at 16 months with an approximate 99% increase in WNK1:WNK4 in aged females vs. an approximate 90% decrease in the WNK1:WNK4 ratio in aged males compared to the respective ratios on NS intake (Fig. 6). In 16-month-old female rats, we observed a loss of dietary sodium-evoked suppression of total SPAK/OxSR1 levels and an increase in serine SPAK/OxSR1 phosphorylation (Fig. 7).

### Impact of the renal sympathetic nerves on age-dependent hypertension and NCC regulatory kinases

**Fig. 8:**
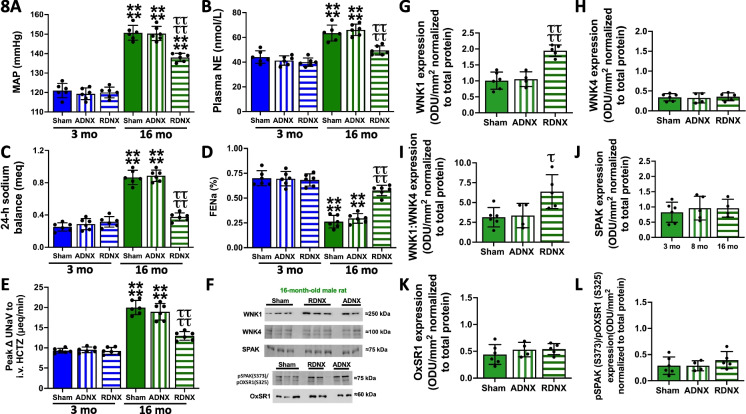
Impact of renal nerve ablation on age-dependent hypertension and NCC regulation. **A** Mean arterial pressure (MAP; mmHg), **B** circulating plasma NE content (nmol/L), **C** 24-h sodium balance (meq), **D** FENa (%), **E** in vivo NCC activity expressed as peak natriuretic response (ΔUNaV) to intravenous hydrochlorothiazide (HCTZ; 2 mg/kg bolus, 2 mg/kg hour infusion), **F** representative immunoblots, **G** total WNK1 expression (optical density units (ODU)/mm^2^ normalized to total protein), **H** total WNK4 expression (ODU/mm^2^ normalized to total protein), **I** WNK1:WNK4 expression ratio, **I** total SPAK expression (ODU/mm^2^ normalized to total protein), **K** total OxSR1 expression (ODU/mm^2^ normalized to total protein), **L** pSPAK (S373)/pOxSR1 (S325) expression (ODU/mm^2^ normalized to total protein) in 3- and 16-month-old male Sprague–Dawley rats maintained on a lifelong normal salt intake (NS; 0.6% NaCl) *N* = 4–6 per group, mean ± SD. MAP, mean arterial pressure; NE, norepinephrine; FENa, fractional excretion of sodium; HCTZ, hydrochlorothiazide; ADNX, selective afferent renal nerve ablation; RDNX, renal denervation. ****P < 0.0001 vs. respective 3-month-old group value. ^τ^*P* < 0.05 vs. 16-month ADNX group value ^ττττ^*P* < 0.0001 vs. 16-month ADNX group value

Bilateral renal denervation (RDNX) and selective sensory ARN ablation were utilized to assess the contribution of the renal sympathetic nerves to age-dependent hypertension in male rats. In both young and aged male rats, control sham renal denervation had no effect on baseline BP, 24-h sodium balance, FENa, sympathetic outflow, or estimated in vivo NCC activity (Fig. 8A–E). In young male rats, validated selective ablation of the sensory ARN (Figure S13) or of both the afferent and efferent renal sympathetic nerves by RDNX (Figure S14) did not alter BP, renal sodium handling, or sympathetic tone (Fig. 8A–E). In 16-month-old male rats, selective ARN ablation did not affect BP, 24-h sodium balance, FENa, NCC activity, or indices of sympathetic tone (Fig. 8A–E). In contrast, bilateral RDNX in 16-month-old male rats significantly lowered BP by approximately 12 mmHg, restored sodium balance to that observed in young rats, significantly attenuated NCC activity, reduced sympathetic outflow, and increased the renal excretory responses to an acute 5% isotonic saline VE (Figs. 8A–E, S24). Selective ARN ablation had no impact on the expression of WNK1, WNK4, SPAK, or OxSR1 and did not alter SPAK/OxSR1 phosphorylation (Fig. 8F–L). As observed with selective ARN ablation, RDNX did not impact the expression or phosphorylation of SPAK or OxSR1 (Fig. 8). While RDNX had no impact on WNK4 levels, we observed a significant increase in WNK1 expression resulting in a significantly increased WNK1:WNK4 ratio.

## Discussion

The major finding of this study is that normal aging evoked the development of hypertension and the salt sensitivity of BP in male, but not female, Sprague–Dawley rats by a mechanistic pathway involving increased sympathetic outflow. The increase in sympathetic tone was driven, in part, by an impaired ARN sympathoinhibitory reno-renal reflex. Elevated sympathetic tone to the kidney evoked the dysregulation of the NCC regulatory kinases WNK1, WNK4, and SPAK to mediate increased NCC activity, renal sodium retention, elevated BP, and the salt sensitivity of BP. Critically, ablation of the renal sympathetic nerves lowered BP, restored sodium balance, and decreased NCC activity via a WNK1:WNK4 pathway. In contrast, female rats did not develop age-dependent hypertension or the salt sensitivity of BP.

### Sex-dependent impact of age on BP and sympathetic tone

Mechanisms of hypertension are typically investigated using young animal models in which genetic or experimental manipulations drive the pathophysiology [8]. In the current studies, we utilized the Sprague–Dawley rat to study the pathophysiology of age-dependent hypertension. In male rats, we observed the development of age-dependent hypertension in which increased BP was associated with increasing age. This finding, in acutely instrumented rats, which have a moderately elevated baseline BP, was replicated and validated in a separate cohort of male rats in which BP was assessed by radiotelemetry. In this radiotelemetered cohort, we observed an age-dependent increase in BP of the same magnitude and time course as seen in acutely instrumented rats—with an approximate 23 mmHg increase in BP between 3- and 16-month-old male rats and an approximate 10 mmHg increase in BP between 3- and 8-month-old rats. Hypertension was accompanied by differential age-related increases in central sympathetic outflow to multiple beds—with only sympathetic outflow to the kidney increasing in a linear manner with age and correlating with the age-dependent increases in BP. It is well established that there is a differential impact of regional alterations in sympathetic outflow on BP and our data supports that there is an age-dependent increase in sympathetic outflow that correlates with increased BP—mimicking the elevations in sympathetic tone and BP observed with age in humans [9, 44]. However, we observed no increase in RAAS activity with age. These data support the male Sprague–Dawley rat as a model of age-dependent hypertension, similar to that in humans, as studies of human aging indicate that systemic RAAS activity does not increase with age [45]. However, we acknowledge that our studies do not preclude the possibility that enhanced sensitivity of the brain RAAS contributes to the observed age-dependent increase in BP. Our data strongly suggest that in these aged rats, there is no impairment of renal function, as assessed by proteinuria, urinary KIM-1 or estimated GFR, or structure. These data are supported by prior findings that male rats do not develop structural or functional renal impairments until 20 months of age [46], and suggest that we are not observing a potentially confounding effect of age-dependent alterations in renal hemodynamics in our studies. In contrast, female rats did not develop age-dependent hypertension or alterations in indices of sympathetic outflow. It should be noted that our data of no change in uterus weight or plasma estradiol levels is suggestive of an absence of the effect of age on ovarian function in this model as would be anticipated owing to the fact that female Sprague–Dawley rats maintain a state of persistent estrous following cycle cessation [47]. Additionally, as detailed in Supplemental Table 4, we observed a significant difference in weight, and weight gain from 3 to 16 months between male and female rats. However, we observed a significant sex-dependent weight difference across the lifespan, with males approximately 50% heavier than aged-matched females at all timepoints studied suggesting that the observed differences in weight are not the primary driver of the observed hypertensive phenotype. Further, the observed sex difference in BP is supported by a plethora of studies in other hypertensive rat models, including the Spontaneously Hypertensive Rat, Dahl Salt Sensitive (DSS), DOCA-salt, and Angiotensin-II-infused rat, in which young male rats had a BP difference of 20 mmHg or more compared to female rats [48].

### Sex-dependent impact of age on renal sodium handling and ARN activity

To investigate the potential mechanisms underlying the observed sex differences in BP, owing to the central role of the kidney in BP regulation and evidence that renal sodium retention is a potential contributor to age-dependent hypertension [49], and the observation of increased 24-h sodium balance in aged male rats, we assessed acute renal sodium handling and the role of the sympathoinhibitory sensory ARN reno-renal reflex. Our approach employed an acute VE, which activates the mechanosensitive sensory ARN [50] (by increasing renal pelvic pressure) and PVN parvocellular sympathoinhibitory neurons [12] to evoke suppression of efferent renal sympathetic outflow [35] and natriuresis (i.e., a sympathoinhibitory reno-renal reflex). An acute VE produced a robust natriuretic and diuretic response in 3-month-old male and female rats. This response was unaffected by age in female rats. In these same animals, consistent with prior studies demonstrating that an acute VE activates sympathoinhibitory parvocellular PVN neurons [40], we observed an increase in the number of Fos-positive cell bodies (a marker of neuronal activation) in all parvocellular PVN subnuclei compared to levels observed pre-volume expansion in female rats. However, the natriuretic and diuretic responses, and the number of Fos-positive PVN cell bodies, were significantly and progressively blunted in 8-month and 16-month-old male rats with the predominant attenuation occurring in the medial parvocellular region that is linked to sympathetic outflow. Our evidence of an age-dependent impairment in renal sodium handling is supported by evidence that aged male Fisher rats exhibit impaired natriuresis in response to an acute VE [51] and that aged dogs have impaired suppression of renal sympathetic nerve activity [52]. Together with our prior study conducted in young male SD rats, which demonstrated that maximal VE-evoked natriuresis and PVN neuron activation requires the presence of the sensory ARN [12], we speculate that the observed age-related reduction in VE-evoked natriuresis and PVN neuron activation reflects an impairment in the mechanosensitive sympathoinhibitory ARN reno-reflex.

To assess the impact of age on ARN responsiveness, the ARN were directly activated in an ex vivo renal pelvis preparation with NE, a general stimulus that activates the ARN via α_1_-adrenoceptors—with no selectivity for mechano- vs. chemosensitive ARN terminals, and 450 mM NaCl, a selective chemoreceptor stimulus near the upper limit of urinary sodium concentration [12]. In support of our VE data, we observed no impact of age on ex vivo ARN responsiveness (assessed as substance P release) in female rats. In contrast, in male rats, aging was associated with reduced responsiveness to NE, but not to 450 mM NaCl, suggesting that the observed impairment in ARN responsiveness is related to the mechanosensitive but not the chemosensitive ARN response—as observed in our in vivo VE study in which renal perfusion pressure is increased to activate the mechanosensitive ARN. Coupled with our data demonstrating no age-dependent alterations in the presence of CGRP (an afferent nerve marker) or CGRP levels in the kidneys of male rats, our data suggest that there is an age-dependent impairment in the sensory sympathoinhibitory ARN pathway, which our data suggests is occurring as a result of impairments in the mechanosensitive arm of the reflex.

### Sex-dependent impact of age and dietary salt intake on BP and NCC activity

In male rats maintained on a lifelong standard rodent diet that underwent an in vivo renal sodium transporter assay, we observed an age-dependent increase in BP, increased NCC protein levels and activity (assessed as estimated in-vivo NCC activity and NCC phosphorylation (which stabilizes NCC in the plasma membrane and increases its intrinsic activity [53])), and no alteration in estimated ENaC activity. In contrast, although young female rats have higher baseline NCC activity than male rats, we observed no age-dependent change in NCC activity despite a moderate increase in total NCC protein expression in normotensive aged female rats. These data provide compelling evidence that alterations in NCC activity contribute to age-dependent elevations in BP independent of those evoked by exogenous Angiotensin-II infusion [54]. Our data that NCC antagonism restored sodium balance and reduced BP, without modulating the acute natriuretic response to a volume expansion, in aged hypertensive male rats strongly suggest a role for the NCC in blood pressure regulation and long-term sodium homeostasis in age-dependent hypertension. These findings are supported by evidence that in hypertensive patients aged 25–65 years, increasing age correlates with an improved therapeutic BP response to thiazide diuretics [55].

In response to a 21-day dietary HS intake (classical experimental paradigm to assess the salt sensitivity of BP in rat), young male and female rats exhibit dietary sodium-evoked suppression of sympathetic tone, estimated ENaC and NCC activity, NCC expression, and NCC phosphorylation—all of which contribute to the maintenance of a salt resistant phenotype. Aged female rats maintain dietary sodium-evoked suppression of sympathetic tone and total NCC expression and remain salt resistant. Given that 16-month-old of female rats suppress total NCC levels, but not overall NCC activity, we believe that in aged rats overall suppression of the NCC system (less total NCC and therefore less NCC to be active) vs. suppression of both total NCC and NCC activity as seen at 3 and 8 months is the major regulator of NCC function. These data suggest that dietary sodium-evoked suppression of sympathetically driven NCC activation contributes to salt resistance in female rats across the lifespan. Given that several female sex hormones, including prolactin, estrogen, and progesterone, are involved in NCC regulation via multiple mechanisms, including actions on the RAAS and central nervous system [56, 57], we speculate that the female sex steroids facilitate NCC regulation with age owing to the fact that female Sprague–Dawley rats maintain a state of persistent estrous following cycle cessation [47] and our observation of no change in uterus weight or plasma estradiol levels that suggests the absence of an effect of age on ovarian function. In contrast, aged male rats exhibit increased sympathetic tone and a failure to downregulate NCC activity and expression in response to dietary salt intake. We hypothesize that this contributes to the observed development of the salt sensitivity of BP. This is supported by evidence that increased sympathetic tone drives NCC activity and expression and the development of salt-sensitive hypertension [18, 19, 58] Further, in contrast to young rats, which exhibit enhanced ARN responsiveness to HS intake, hypertensive 8- and 16-month-old male rats fail to increase ARN responsiveness to a NE stimulus. Additionally, as observed on a normal salt intake, 16-month-old male rats on a HS diet exhibit a blunted renal response to VE (mechanosensitive ARN stimulus) but no elevation in blood pressure—demonstrating intact control of total peripheral resistance and a central role of the kidney and renal sodium handling in the development of the salt sensitivity of BP. We speculate that this failure to enhance sympathoinhibitory ARN reno-reflex activity during HS intake, as we have previously documented in DSS rats, may be a common pathological feature underlying the pathophysiology of the salt sensitivity of BP that drives sympathetically mediated increases in NCC activity [12]. While not investigated, given that we saw no age-dependent alterations in the RAAS in hypertensive males, we speculate, based off prior reports that testosterone predominantly drives sodium reabsorption via increased activity of the RAAS system [59, 60], that testosterone levels are not driving the observed physiological phenotypes.

### Sex-dependent impact of age and dietary salt intake on NCC regulatory kinases

Considering the divergent sex-dependent regulation of the NCC with age and in response to dietary salt intake, we investigated the expression and activity of NCC regulatory kinases. Both male and female rats exhibit increased expression of WNK1 at 16 months and decreased WNK4 expression at 8 and 16 months. This resulted in an altered ratio of WNK1 to WNK4 expression in both sexes. We observed an increase in SPAK expression in aged male, but not female rats, and no change in OxSR1 levels with age irrespective of sex, supporting prior studies that OxSR1 has a relatively minor contribution to NCC regulation [61, 62]. In relation to SPAK and OxSR1 activity, we observed no age-dependent changes in pSPAK(T233)/pOxSR1(T185) in male or female rats, and a significant decrease in pSPAK(S373)/pOxSR1(S325) in16-month-old male rats only. We speculate that this decrease in pSPAK/OxSR1 reflects a compensatory response to reduce NCC translocation to the membrane to counteract increased SPAK expression with age. Given that in vitro studies have demonstrated the existence of WNK heteromultimers that may result in an additive, subadditive, or synergistic effect on NCC depending on the ratio of WNK kinases to SPAK/OxSR1 [20], our data suggest a central role for SPAK in NCC regulation in age-dependent hypertension in male rats that may involve WNK heterodimers.

Our data reveal sex- and age-dependent regulation of WNKs in response to HS intake. In young male rats, we observed a selective dietary sodium-evoked suppression of WNK1 compared to a selective suppression of WNK4 in female rats. A sex-dependent differential WNK response to dietary salt intake was maintained across the lifespan, with female, but not male rats, exhibiting a dietary salt-evoked increase in the WNK1:WNK4 expression ratio compared to that observed on a normal salt intake at all ages. In contrast, we observed a dietary salt-evoked decrease in the WNK1:WNK4 ratio in 16-month-old male rats. We have previously observed the dynamics of an increased WNK1:WNK4 expression ratio in the DSR rat that was absent in the DSS rat [19], which exhibits increased renal sympathetic tone and increased NCC expression and activity as observed in aged male rats. Collectively, these data suggest a pivotal role of the dynamic expression of WNK1 to WNK4 in NCC regulation and the salt sensitivity of BP. Further, in response to HS intake, we observed downregulation of total SPAK and reduced SPAK/OxSR1 phosphorylation in male and female 3-month-old rats, which was accompanied by reduced NCC activity. In aged female rats, which exhibit no dietary sodium-evoked increase in NCC activity during HS intake, we observed a loss of dietary sodium evoked-suppression of total SPAK levels and a moderate increase in serine SPAK/OxSR1 phosphorylation. In contrast, in aged male rats on a HS diet, which also fail to suppress total SPAK and OxSR1 levels, we observed a failure to suppress SPAK/OxSR1 phosphorylation at 16 months. These data suggest that total SPAK expression levels versus SPAK phosphorylation has a significant impact on NCC activity with age on a normal salt diet given comparable SPAK/OxSR1 phosphorylation in aged male and female rats with increased total SPAK and NCC activity in aged males only. A central role of increased pSPAK/pOxSR1 in the salt sensitivity of BP in aged males is suggested and this is supported by increased (~ sixfold) pSPAK/OxSR1 in DSS rats, which corresponds to increased NCC phosphorylation [19]. Collectively, our data, which is correlative in nature, suggest that increased SPAK/OxSR1 phosphorylation, which may be modulated by WNK heterodimers, is a major contributor to increased NCC phosphorylation and activity, and the salt sensitivity of BP in aged male, but not female, rats.

### Differential impact of ARN ablation versus RDNX on age-dependent hypertension in male rats

Supporting our central hypothesis, that the sympathoinhibitory afferent renal nerve reno-renal reflex is impaired in aged hypertensive male rats, selective ablation of the ARN did not lower BP and sympathetic tone, improve renal sodium handling, reduce NCC activity, or impact the expression of NCC regulatory kinases in aged male rats. In contrast, renal denervation lowered BP, reduced central sympathetic outflow, reduced NCC activity, restored sodium balance, increased the natriuretic response to an acute VE, and selectively increased WNK1 expression and the WNK1:WNK4 ratio in 16-month-old rats. Renal denervation, which lowered blood pressure significantly, did not return blood pressure to baseline levels, indicating other mechanisms, in addition to increased renal sympathetic tone, contribute to the observed age-dependent elevations in blood pressure (e.g., arterial stiffness, inflammation). Our data that selective ARN ablation did not evoke hypertension in young rats is supported by our prior study [12] and a recent study in which a small, but significant, increase in BP (approximately 5 mmHg) was observed 10 days post ARN ablation by radiotelemetry suggesting that under baseline conditions the ARN contribute to BP regulation but are not essential to maintain normotension. In contrast, our present data, which demonstrate the impact of attenuated ARN reflexes with age, suggest a pivotal role of the ARN in BP regulation with age and the salt sensitivity of BP. This hypothesis is supported by our prior study that validated (biochemically and pharmacologically) selective ARN ablation immediately prior to the placement of animals on a HS intake evokes a rapid and sustained increase in BP (i.e., the salt sensitivity of BP) and prevents global and renal sympathoinhibition in young male salt-resistant rat phenotypes (Sprague–Dawley and Dahl Salt Resistant) [12]. We acknowledge that in Foss et al. (2015), ARN ablation did not increase BP during HS intake in male Sprague–Dawley rats [29]. However, there are several critical differences between our prior work and Foss et al., which utilized (1) 14-days post ARN ablation prior to HS challenge during which time (a) renal reinnervation is in progress and (b) ARN-independent adaptive compensatory mechanisms, (2) a different experimental paradigm of a stepped increased in HS intake over 7 weeks, and (3) no functional assessment of ARN ablation. Providing additional support for a role of the ARN in BP regulation, the ARN is involved in the maintenance of elevated BP in the DOCA-salt hypertension model [16]. Collectively, our data suggest that the recent FDA-approved approach of renal denervation represents a therapeutic approach in hypertensive patients with impaired ARN reflexes, particularly within the growing aged population, to modulate sympathetic outflow, renal sodium handling, and NCC activity and lower blood pressure.

### Study limitations

Although our Fos data suggest that there is reduced VE-evoked activation of PVN parvocellular neurons with age, we acknowledge that Fos immunostaining does not always equate to neuronal activation and that our approach does not confirm that the identified neurons are functionally activated or possess a sympathoinhibitory phenotype as double labeling for tyrosine hydroxylase or oxytocin/vasopressin was not conducted. A minor technical limitation of this study is the direct measurement of BP and renal excretory function following acute instrumentation of the animals in the majority of studies. However, our validation of our blood pressure phenotype in male rats by radiotelemetry both replicates and validates our findings and our approach of acute instrumentation remains the sole means of assessing BP and in vivo physiological activity of the NCC within the same animal. Further, the use of acute instrumentation, which has been previously used by multiple laboratories including our own, to assess the renal excretory responses to VE, increases the reproducibility and rigor of our current and previously published data. The observed sex-dependent differences in multiple parameters, including BP, renal sodium handling, and NCC activity, warrant further investigation into the mechanistic basis of these differences, such as the effects of sex hormones on these factors and post-translational modifications, including ubiquitylation, relevant to NCC activity, but beyond the scope of these studies. We acknowledge it is a limitation that cycle state of females was not analyzed and that estradiol levels were assessed by ELISA versus the use of high-sensitivity gas chromatography-tandem mass spectrometry, but as our data reproduce prior findings, we believe our approach is valid. It is also a limitation that the female rat phenotype used in these studies does not reproduce the hypertensive phenotype of women—in which there is greater salt sensitivity of BP versus men regardless of age, BP, or menopause status.[63] Additionally, as these studies were conducted in animals purchased exclusively from Envigo, it remains to be established if Sprague–Dawley rats from other sources (e.g., Taconic, Charles Rivers) develop age-dependent hypertension.

## Supplementary Information

Below is the link to the electronic supplementary material.

Supplementary file1 (PDF 22006 KB)

## Data Availability

Original data is available upon reasonable request.
