# Supplementary material for: Integrated renal and sympathetic mechanisms underlying the development of sex- and age-dependent hypertension and the salt sensitivity of blood pressure
Source: GeroScience. 2024 Jul 8;46(6):6435–58. doi: 10.1007/s11357-024-01266-1 (PMC11494650; doi:10.1007/s11357-024-01266-1)
Supplement: Supplementary file 1 — Supplementary file1 (PDF 22006 KB) [file 11357_2024_1266_MOESM1_ESM.pdf]

Online Supplemental Material

Title: Integrated renal and sympathetic mechanisms underlying the development of sex- and age-dependent hypertension and the salt sensitivity of blood pressure

Short Title: Mechanisms of age dependent hypertension

Authors: Alissa A. Frame[1*], PhD; Kayla M. Nist[2*], MS; Kiyoung Kim,[1] PhD; Franco Puleo[1], PhD; Jesse D. Moreira[4], PhD; Hailey Swaldi, BS, James McKenna 3rd [3], MS, MBA, and Richard D. Wainford,[1,3] PhD

Author Affiliations:

[1]Department of Pharmacology & Experimental Therapeutics and the Whitaker Cardiovascular Institute, Boston University Chobanian & Avedisian School of Medicine, Boston, Massachusetts, USA.
[2]Department of Anatomy & Neurobiology, Boston University Chobanian & Avedisian School of Medicine, Boston, Massachusetts, USA.
[3]Division of Cardiology, Emory University School of Medicine, Atlanta, Georgia, USA.
[4]Department of Health Sciences, Sargent College, Boston University, Boston, Massachusetts, USA.

* These authors contributed equally to this work.

Corresponding Author: Richard D. Wainford, BSc, Ph.D., F.A.H.A., Professor, Emory University School of Medicine, Division of Cardiology, 1750 Haygood Drive, N220, Atlanta, Georgia 30322. Phone: 404-727-3754 Fax: 404-713-0070; E-mail: rwainfo@emory.edu

Supplemental Tables

Primary Antibody	Dilution	Source
NCC (Sodium Chloride Cotransporter)	1:1000	Millipore, Billerica, MA; Cat. No. AB3553
Phosphorylated NCC Thr-53	1:1000	Phosphosolutions, Aurora, CO; Cat. No. p-1311-53
WNK1 (With no lysine kinase 1)	1:200	Santa Cruz, Dallas, TX; Cat. No. 28897
WNK4 (With no lysine kinase 4)	1:1000	Novus Biologicals, Centennial, CO; Cat. No. NB600-284SS
OxSR1 (Oxidative stress response 1)	1:2000	Abcam, Cambridge, MA; Cat. No. ab125468
SPAK (STE20/SPS1-related proline-alanine-rich protein kinase)	1:500	Abcam, Cambridge, MA; Cat. No. ab79045
Anti-phospho SPAK (Ser373)/phosphor-OSR1 (Ser325)	1:500	Millipore Sigma, Burlington, MA; Cat. No. 07-2273
Anti-phospho OxSR1 (T185)/SPAK (T233)	1:500	Abcam, Cambridge, MA; Cat. No. ab 138655
**Secondary Antibody**	**Dilution**	**Source**
HRP (Horseradish peroxidase) donkey anti-rabbit IgG	1:2000	Abcam, Cambridge, MA; Cat. No. ab16284
**Loading Control**	**Concentration**	**Source**
WNK4	0.5 and 1μg	Abnova, Taiwan; Cat. No. P5665
SPAK	0.5 and 1μg	Abcam, Cambridge, MA; Cat. No, ab107696
OxSR1	0.5 and 1μg	US Biological, Salem, MA; Cat. No. 218418

Table S1 Antibodies and loading controls used for immunoblotting

Study Group	Baseline Na^+ excretion (µeq/min)	Baseline K^+ excretion (µeq/min)	Baseline urine output (µl/min)
3-month male	2.34±0.36	2.08±0.34	20.5±3.6
8-month male	2.33±0.29	2.16±0.28	22.1±3.2
16-month male	2.51±0.39	2.16±0.42	20.4±4.2
3-month female	2.46±0.28	2.14±0.37	21.3±4.4
8-month female	2.24±0.30	1.96±0.32	20.7±3.9
16-month female	2.53±0.34	2.05±0.28	21.6±4.1

Table S2 Baseline renal excretory parameters during acute VE assay Baseline sodium excretion (µeq/min), baseline potassium excretion (µeq/min), and baseline urine output (µl/min) during the control isotonic saline infusion period of the acute VE assay in conscious naive male and female 3-, 8- and 16-month-old Sprague-Dawley rats maintained on a lifelong normal (0.6% NaCl) diet. Data are expressed as mean ± SD (N=6/group).

Study Group	Baseline Na^+ excretion (μeq/min)	Baseline K^+ excretion (μeq/min)	Baseline urine output (μl/min)
3-month male NS	2.34±0.36	2.08±0.34	20.5±3.6
3-month male HS	2.33±0.29	2.16±0.28	22.1±3.2
8-month male NS	2.51±0.39	2.16±0.42	20.4±4.2
8-month male HS	2.46±0.28	2.14±0.37	21.3±4.4
16-month male NS	2.24±0.30	1.96±0.32	20.7±3.9
16-month male HS	2.53±0.34	2.05±0.28	21.6±4.1
3-month male NS	2.34±0.36	2.08±0.34	20.5±3.6
3-month female HS	2.33±0.29	2.16±0.28	22.1±3.2
8-month female NS	2.51±0.39	2.16±0.42	20.4±4.2
8-month female HS	2.46±0.28	2.14±0.37	21.3±4.4
16-month female NS	2.24±0.30	1.96±0.32	20.7±3.9
16-month female HS	2.53±0.34	2.05±0.28	21.6±4.1

Table S3 Baseline renal excretory parameters during acute renal sodium transporter assay Baseline sodium excretion (μeq/min), baseline potassium excretion (μeq/min), and baseline urine output (μl/min) during the control isotonic saline infusion period of the acute renal sodium transporter activity assay in conscious naive male and female 3-, 8- and 16-month-old Sprague-Dawley rats maintained on a lifelong normal salt (NS: 0.6% NaCl) diet and then challenged with a NS a high-salt (4% NaCl) diet for 21 days. Data are expressed as mean ± SD (N=5 or 6/group).

Study Group	Bodyweight (g)
3-month male	300±3
8-month male	524±53****
16-month male	545±25*****
3-month female	214±9τ
8-month female	287±12**ττττ
16-month female	301±29**ττττ

Table S4 Bodyweight of male and female Sprague-Dawley rats with age Bodyweight (g) in conscious naive male and female 3-, 8- and 16-month-old Sprague-Dawley rats maintained on a lifelong normal (0.6% NaCl) diet. Data are expressed as mean ± SD (N=6/group). **P<0.01 vs. respective 3-month-old group value. ****P<0.0001 vs. respective 3-month-old group value. ττττP<0.01 vs. respective male age-matched group value.

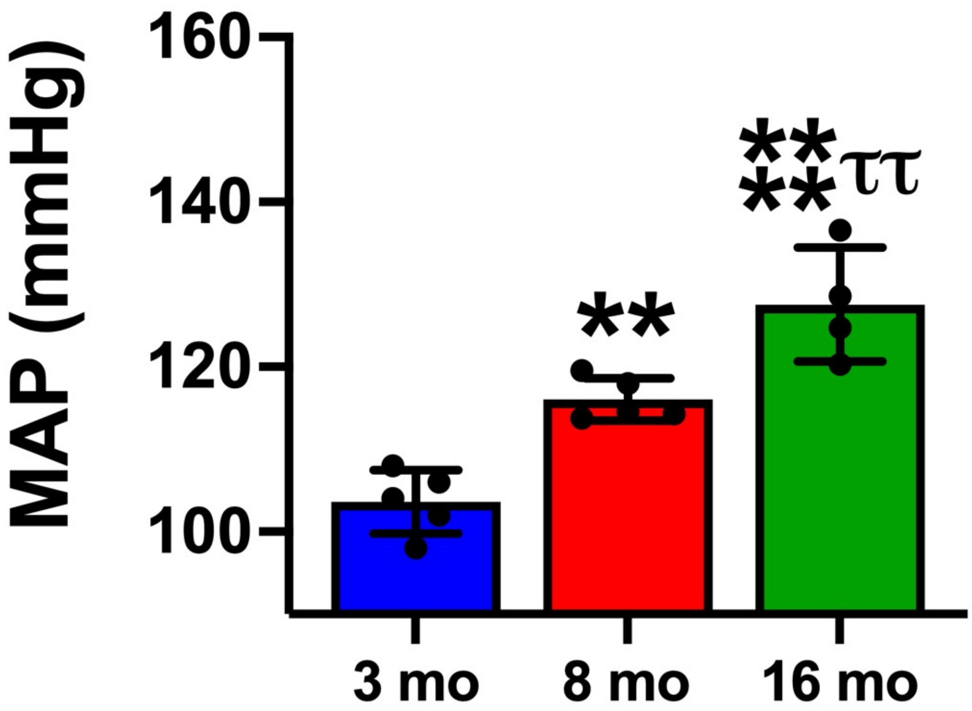

Figure S1 Mean arterial pressure (MAP; mmHg) assessed by radiotelemetry in 3-, 8-, and 16-month-old male Sprague-Dawley rats maintained on a lifelong normal salt intake (NS; 0.6% NaCl). N=4-5 per group, mean ± SD. MAP = mean arterial pressure. **P<0.01 vs. 3-month-old group value. ****P<0.0001 vs. 3-month-old group value. ττP<0.01 vs. 8-month-old group value.

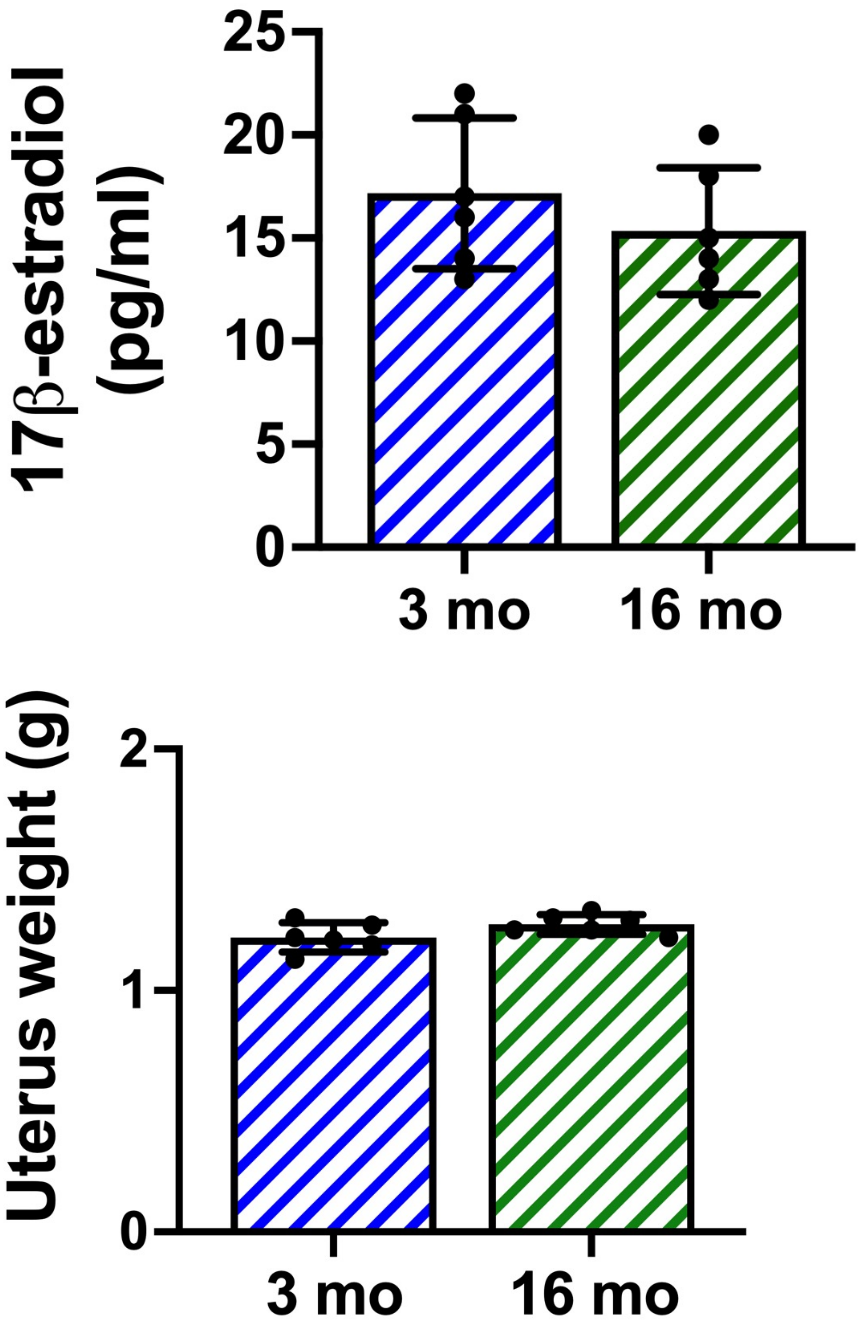

Figure S2 Plasma 17β-estradiol levels (pg/ml) and uterus weight (g) in 3- and 16-month-old female Sprague Dawley rats (N=6/group ±SD).

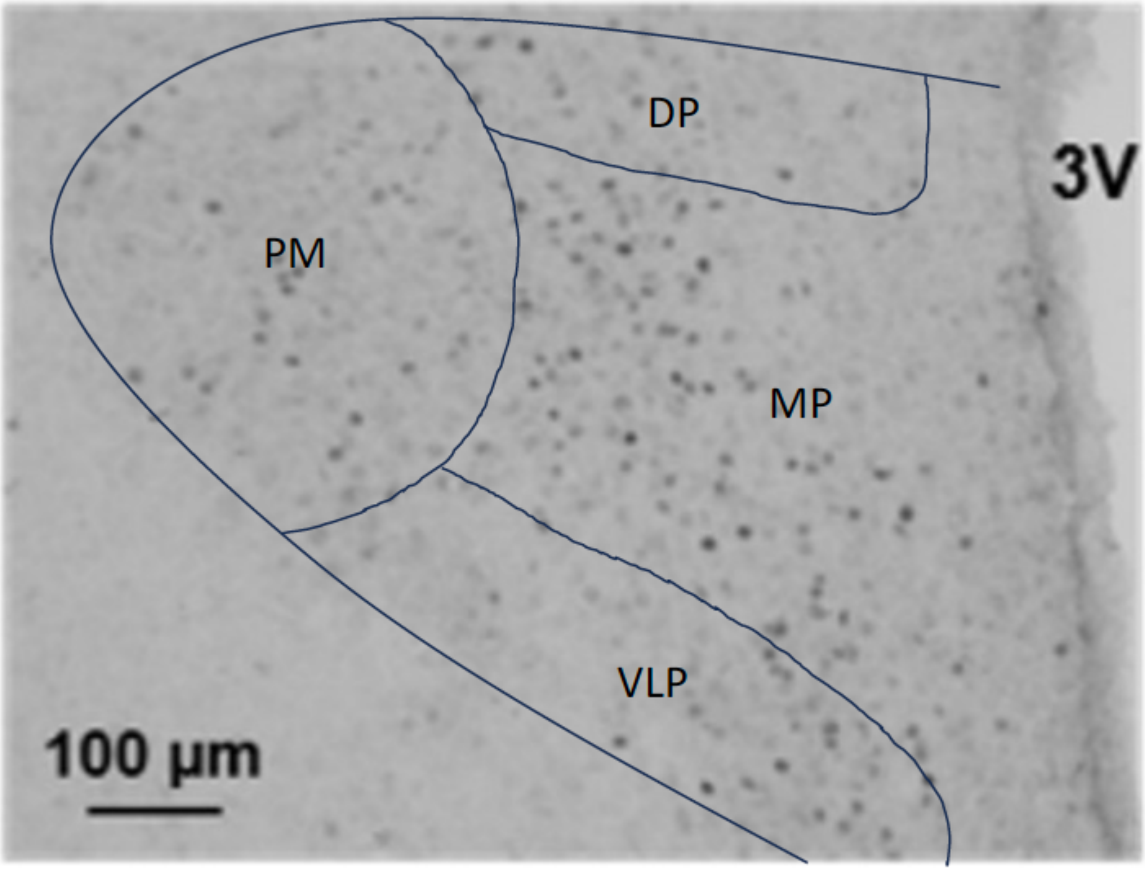

Figure S3 Representative image of level 2 of the PVN from Figure 2 illustrating the boundaries used to identify PVN subregions. DP= dorsal parvocellular; MP= medial parvocellular; VLP= ventrolateral parvocellular; PM= magnocellular.

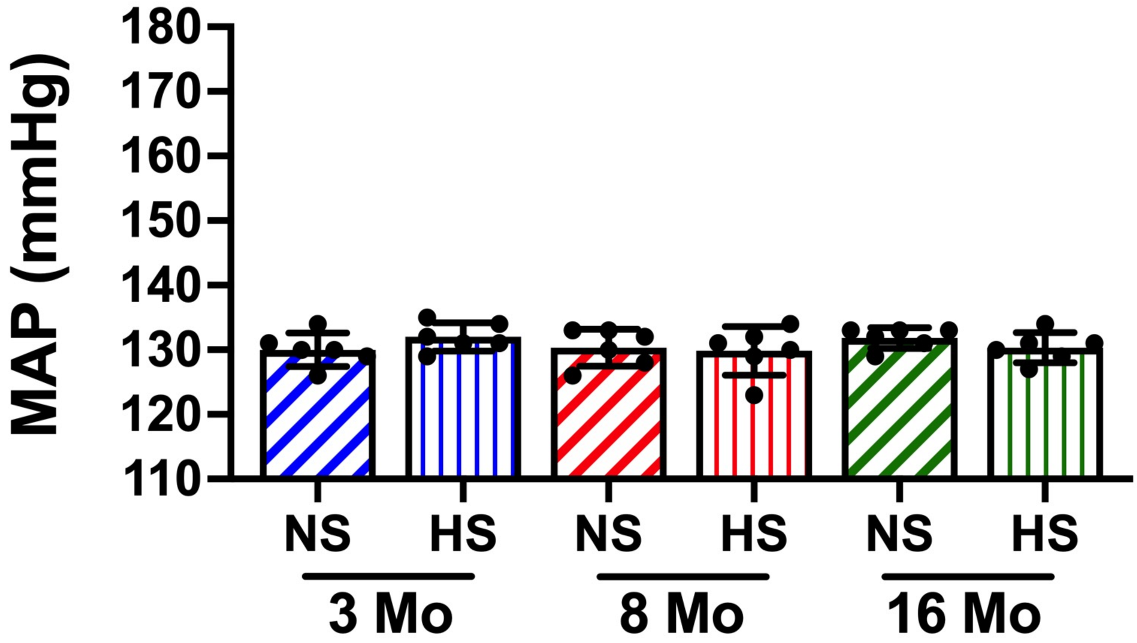

Figure S4 Mean arterial pressure (MAP; mmHg) in 3-, 8-, and 16-month-old female Sprague-Dawley rats maintained on a lifelong normal salt intake (NS; 0.6% NaCl). N=6 per group, mean ± SD. MAP = mean arterial pressure.

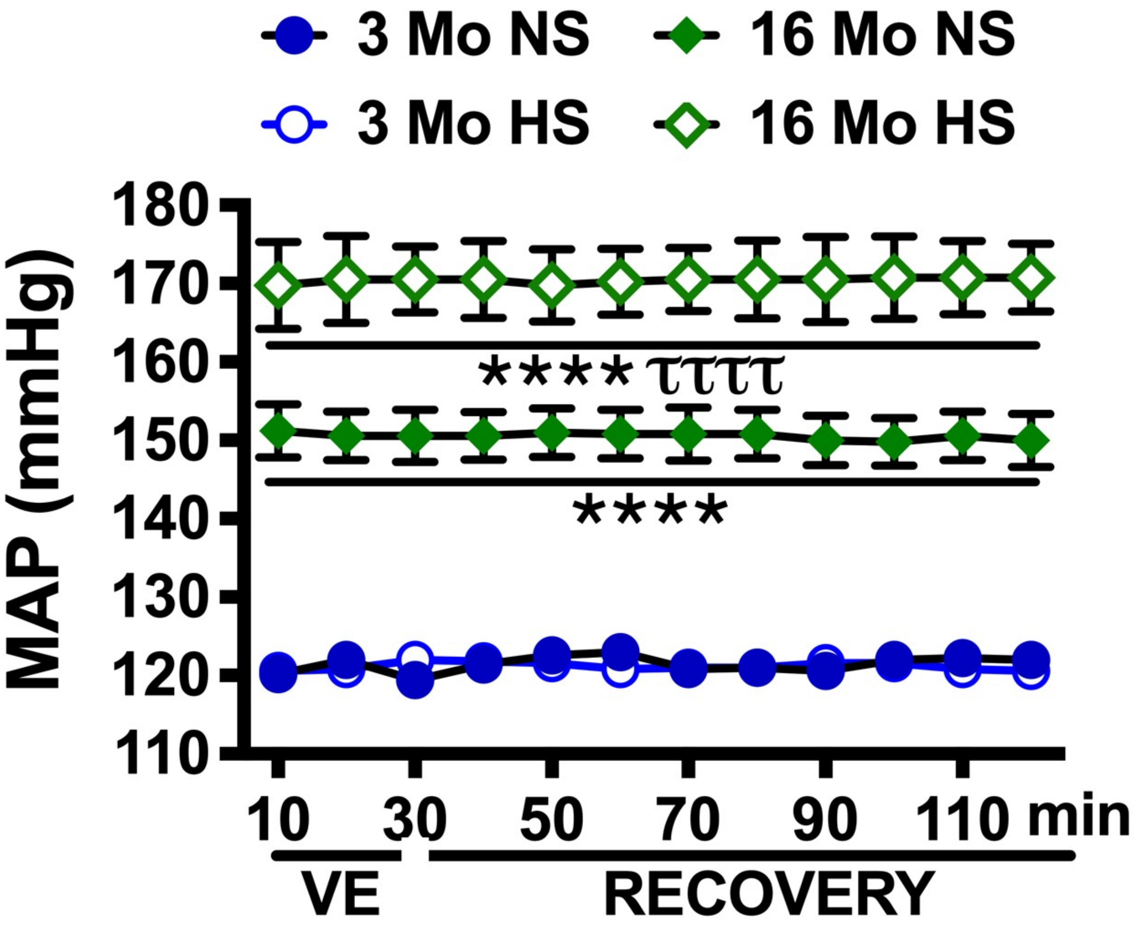

Figure S5 Mean arterial pressure (MAP; mmHg) 3- and 16-month-old male Sprague-Dawley rats during a 5% isotonic saline volume expansion (for which renal excretory data is presented in Figure 4) maintained on a lifelong normal salt intake (NS; 0.6% NaCl) and then challenged with a 21-day NS or high salt (HS; 4% NaCl) diet. N=6 per group, mean ± SD. MAP = mean arterial pressure. ****P<0.0001 vs. respective within diet 3-month-old group value. ττττP<0.0001 vs. respective 8-month-old group value.

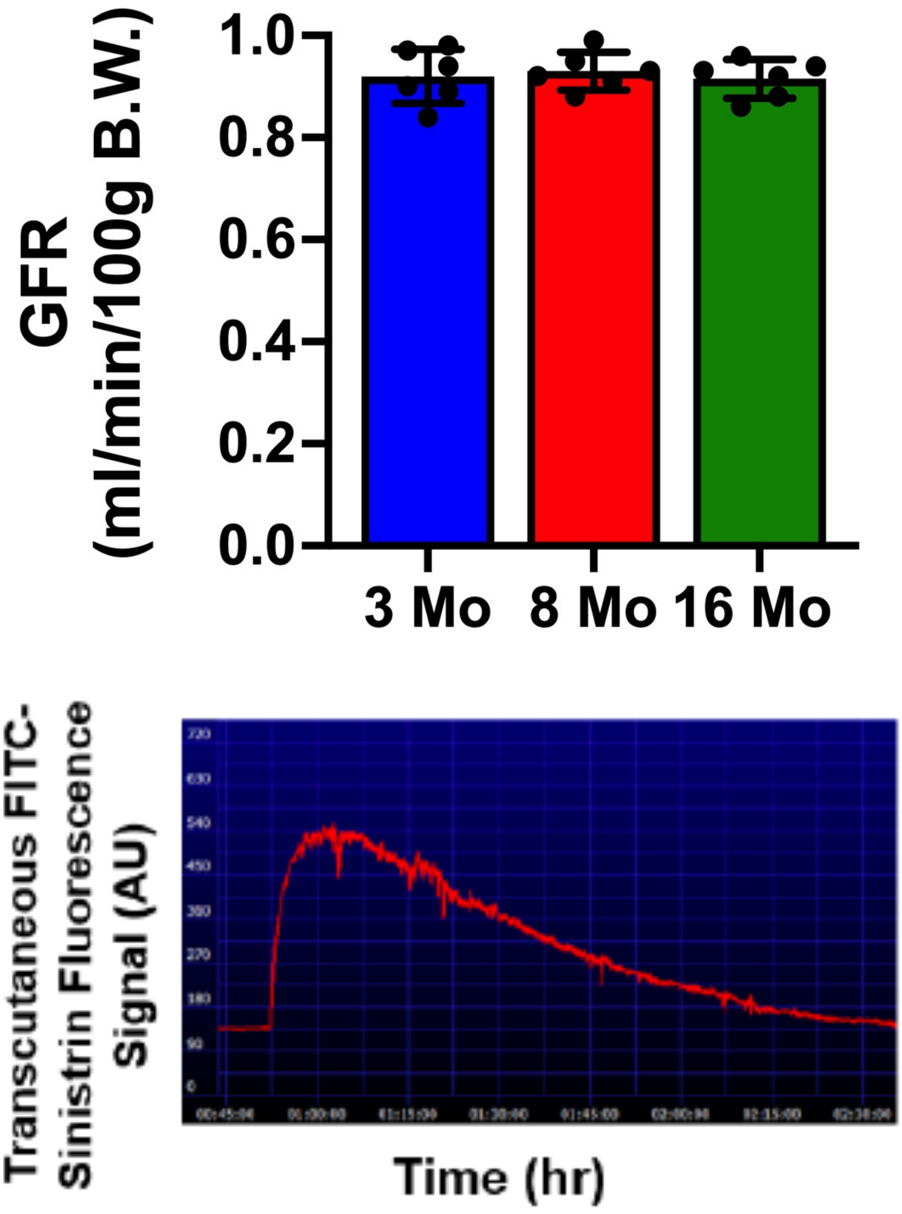

Figure S6 Estimated Glomerular Filtration Rate (GFR: ml/min/100g B.W.) in 3-, 8-, and 16-month-old male Sprague-Dawley rats maintained on a lifelong normal salt intake (NS; 0.6% NaCl) and a representative tracing of the transcutaneous FITC-Sinistrin Fluorescence single (AU; absorbance units). N=6 per group, mean ± SD.

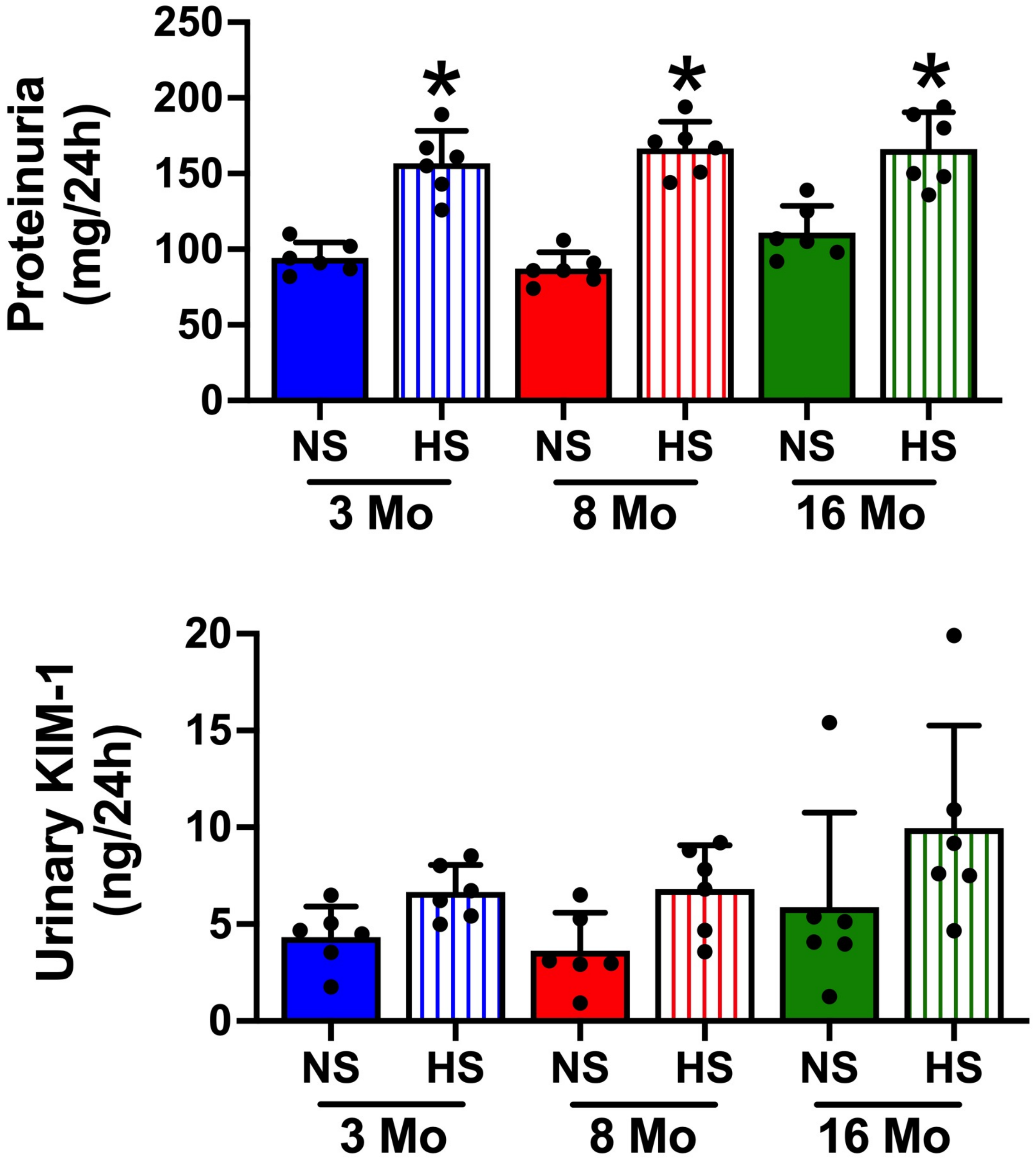

Figure S7 Proteinuria (mg/24) and Urinary KIM-1 (ng/24h) in 3-, 8-, and 16-month-old male Sprague-Dawley rats maintained on a lifelong normal salt intake (NS; 0.6% NaCl) or a 21-day high salt intake (HS; 4% NaCl). N=6 per group, mean ± SD. *P<0.05 vs. respective age-matched NS value.

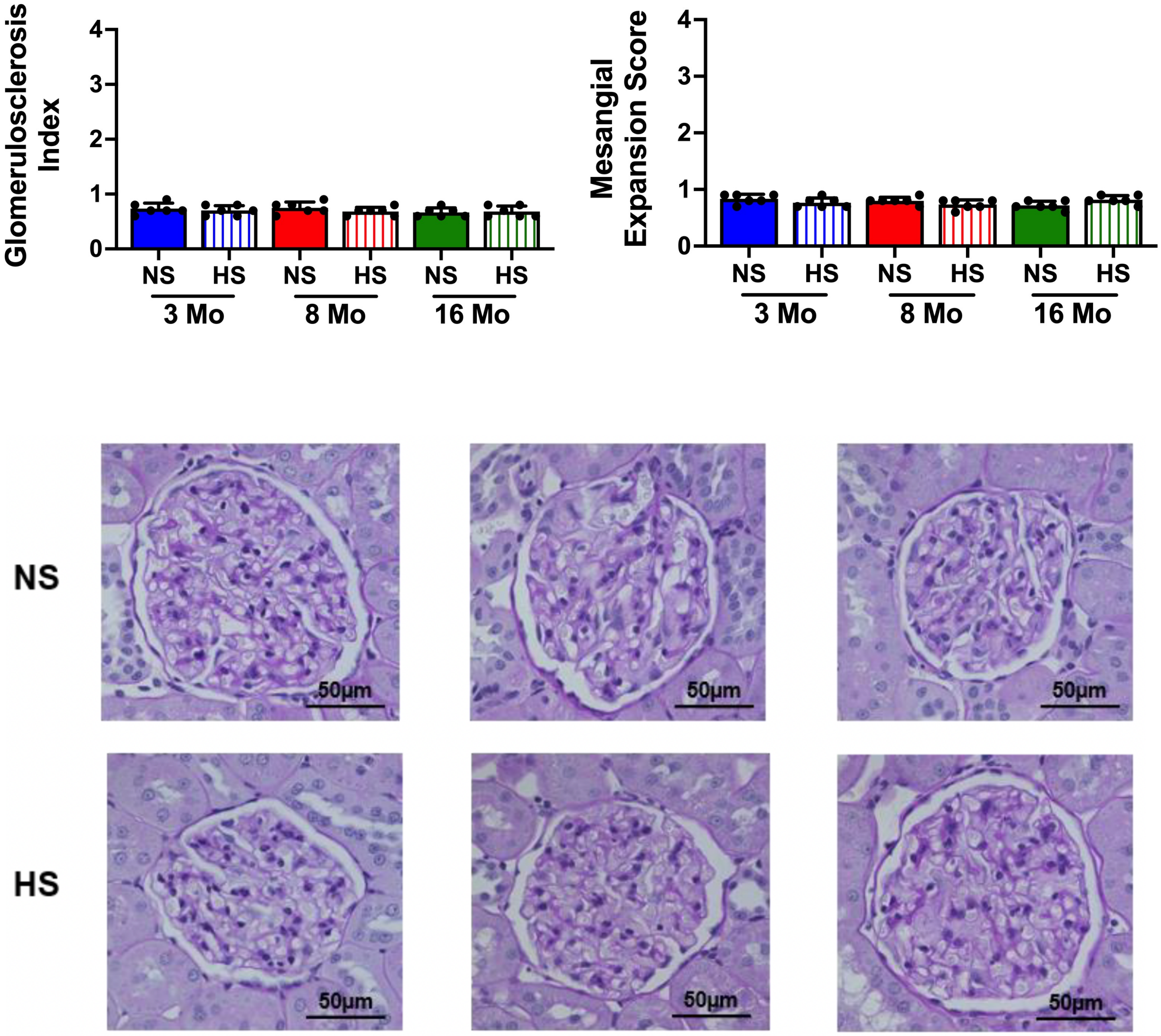

Figure S8 Glomerulosclerosis index and Mesangial expansion score based on semiquantitative assessment of both measures in periodic acid Schiff-stained renal tissue obtained from 3-, 8-, and 16-month-old (Mo) male Sprague-Dawley rats maintained on a lifelong normal salt intake (NS; 0.6% NaCl) or a 21-day high salt intake (HS; 4% NaCl) and representative 20x images from each treatment group. N=6 per group, mean ± SD. 42 glomeruli were assessed per rat for both glomerulosclerosis and mesangial expansion.

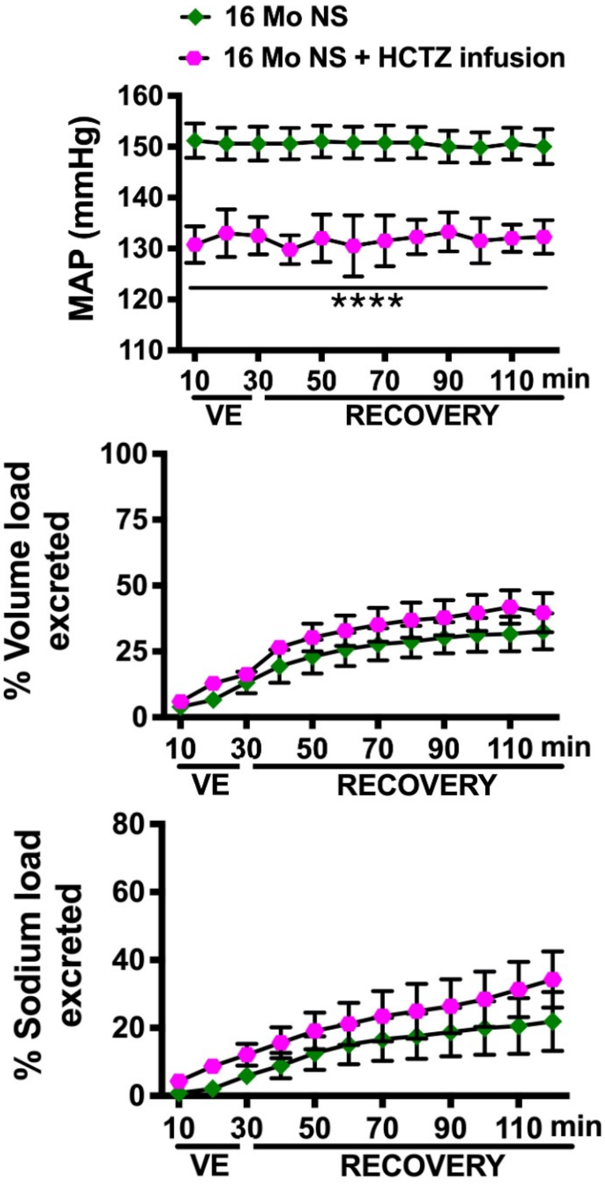

Figure S9 Mean arterial pressure (MAP; mmHg), urinary volume (% volume load excreted) and urinary sodium excretion (% sodium load excreted) in response to a 30-min isotonic saline volume expansion (VE) of 5% body weight followed by a 90-min recovery period in conscious 16-month-old male Sprague-Dawley rats and conscious 16-month-old male Sprague-Dawley rats receiving a 14-day s.c. HCTZ infusion (HCTZ; 4mg/kg/day) maintained on a lifelong normal salt intake (NS; 0.6% NaCl), N=6 per group mean ± SD. MAP = mean arterial pressure. *P< 0.05 vs. respective16-month-old group control value.

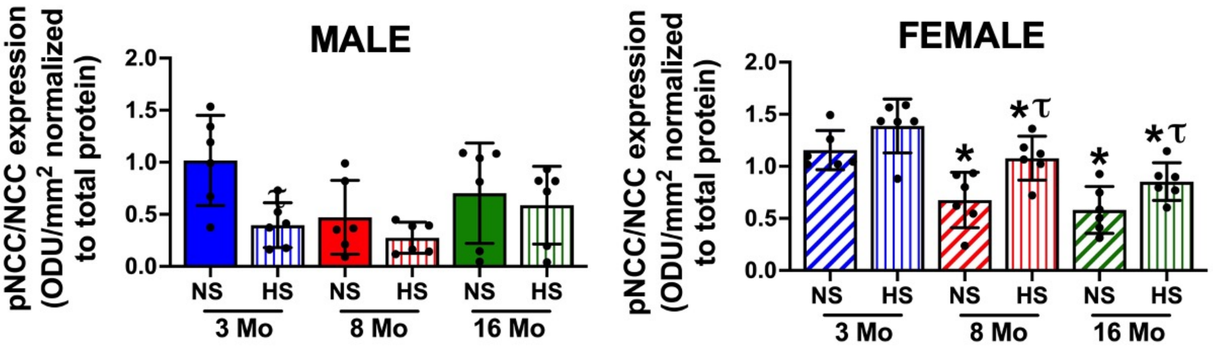

Figure S10 pNCCT53 expression (ODU/mm^2 normalized to total protein) divided by total NCC expression (optical density units (ODU)/mm^2 normalized to total protein) in 3-, 8-, and 16-month-old male and female Sprague-Dawley rats maintained on a lifelong normal salt intake (NS; 0.6% NaCl) and challenged for 21 days with a NS or high salt intake (HS; 4% NaCl), N=6 per group, mean $\pm$ SD. *P<0.05 vs. respective within diet 3-month-old group, τP<0.05 vs. respective within age NS group.

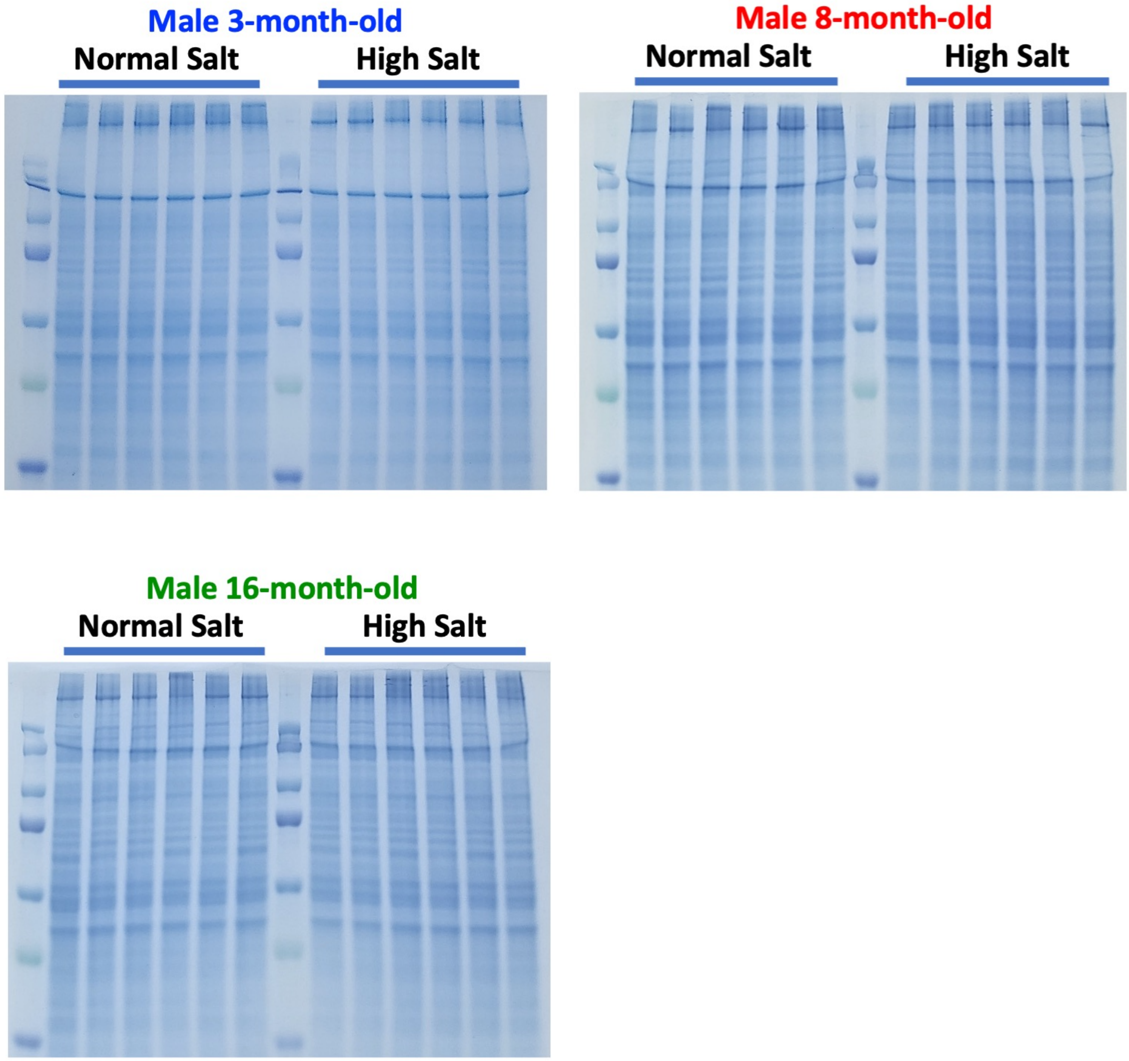

Figure S11 Coomassie blue stain of total protein from 3-, 8- and 16-month-old male Sprague-Dawley rat samples for which immunoblotting data is presented in Figures 5,6 and 7. Left and middle lanes contain protein standard, left side of panel represents total protein loaded after Coomassie stain in rats fed a 21-day normal salt (NS; 0.6% NaCl) and right side a high salt (HS; 4% NaCl) diet.

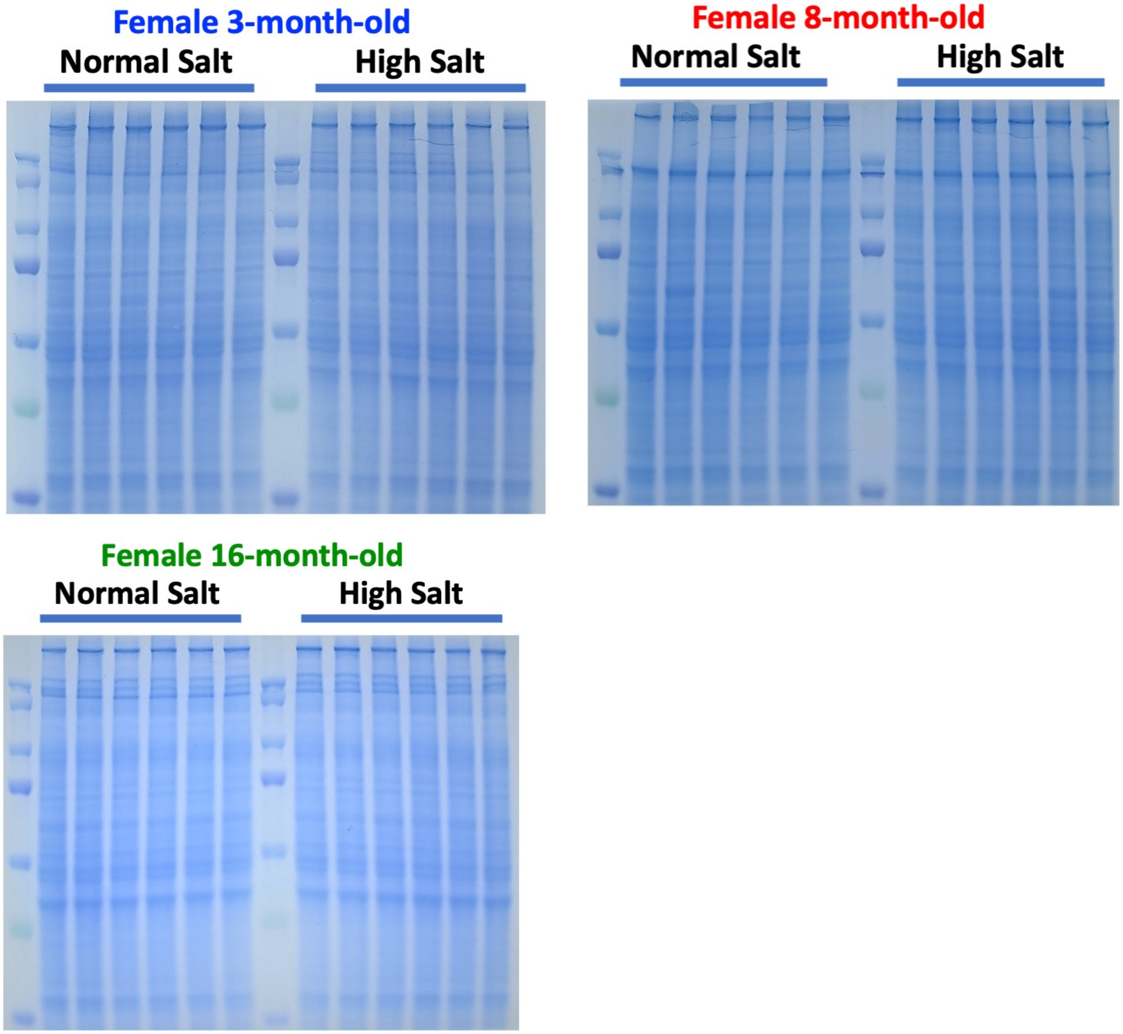

Figure S12 Coomassie blue stain of total protein from 3-, 8- and 16-month-old female Sprague-Dawley rat samples for which immunoblotting data is presented in Figures 5,6 and 7. Left and middle lanes contain protein standard, left side of panel represents total protein loaded after Coomassie stain in rats fed a 21-day normal salt (NS; 0.6% NaCl) and right side a high salt (HS; 4% NaCl) diet.

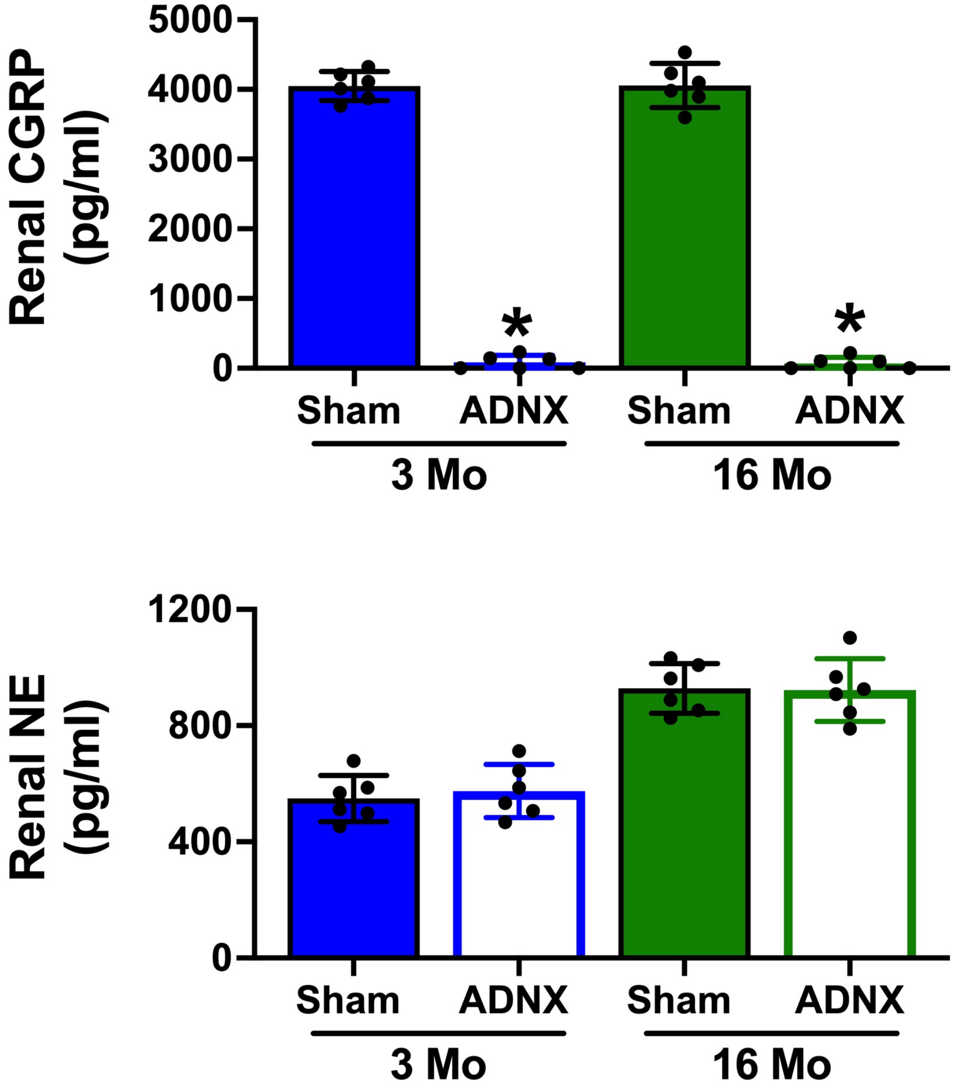

Figure S13 Impact of afferent renal nerve denervation (ADNX) on renal pelvic CGRP and renal NE content (pg/ml) in 3- and 16-month-old male Sprague Dawley rats 14 days after sham surgery or ADNX (N=6/group ±SD) **P* < 0.05 *vs*. respective sham ADNX group value.

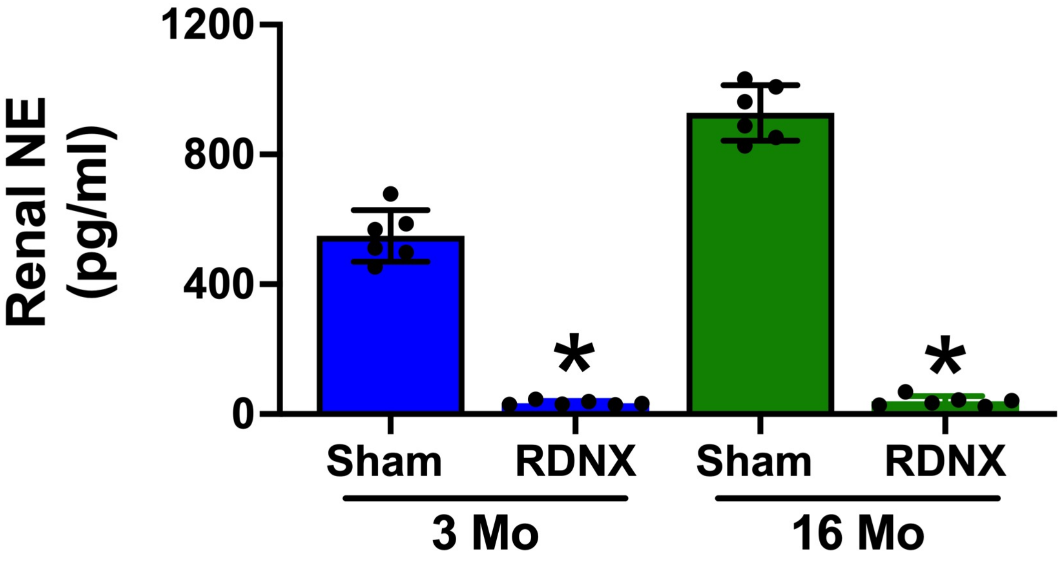

Figure S14 Impact of bilateral renal nerve denervation (RDNX) on renal NE content (pg/ml) in 3- and 16-month-old male Sprague Dawley rats 14 days after sham surgery or RDNX (N=6/group ±SD) *P < 0.05 *vs*. respective sham RDNX group value.

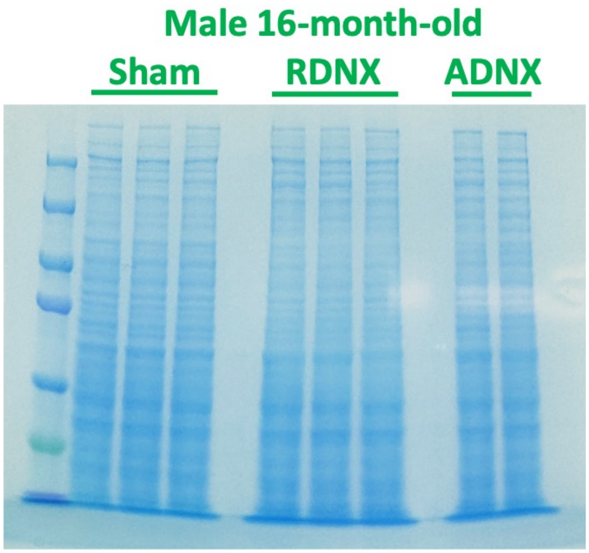

Figure S15 Coomassie blue stain of total protein from 16-month-old male Sprague-Dawley rat samples for which immunoblotting data is presented in Figure 8. Left lane contains protein standard, remaining lanes represent total protein loaded after Coomassie stain in rats fed a lifelong normal salt (0.6% NaCl) intake than underwent sham renal ablation surgery, renal denervation surgery (RDNX) or selective afferent renal nerve ablation (ADNX).

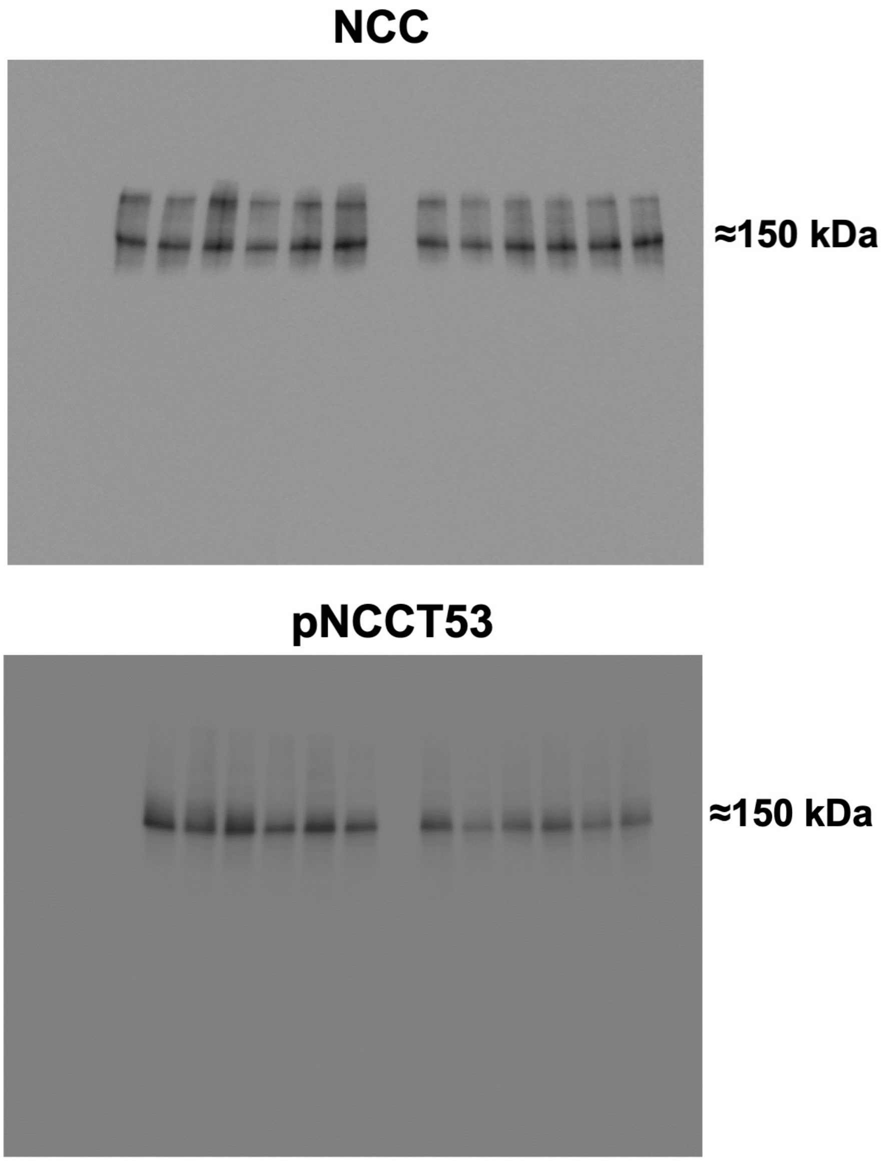

Figure S16 Full representative uncropped blots for NCC and pNCCT53 of images used in Figure 5

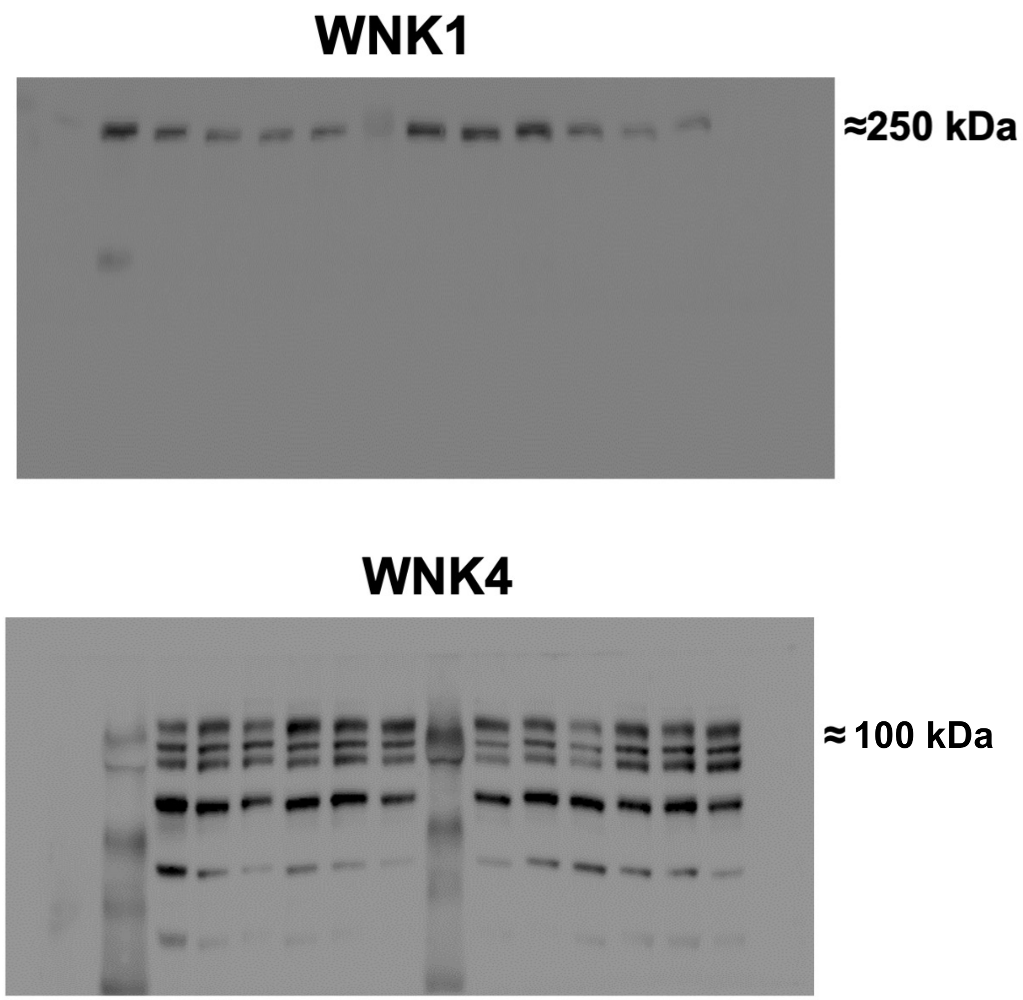

Figure S17 Full representative uncropped blots for WNK1 and WNK4 (top band at approximately 100 kDa used for analysis) of images used in Figure 6

SPAK

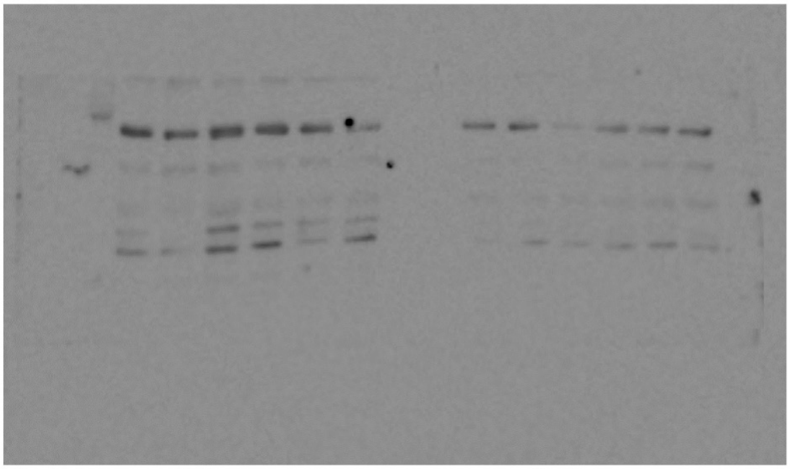

≈75 kDa

OXSR1

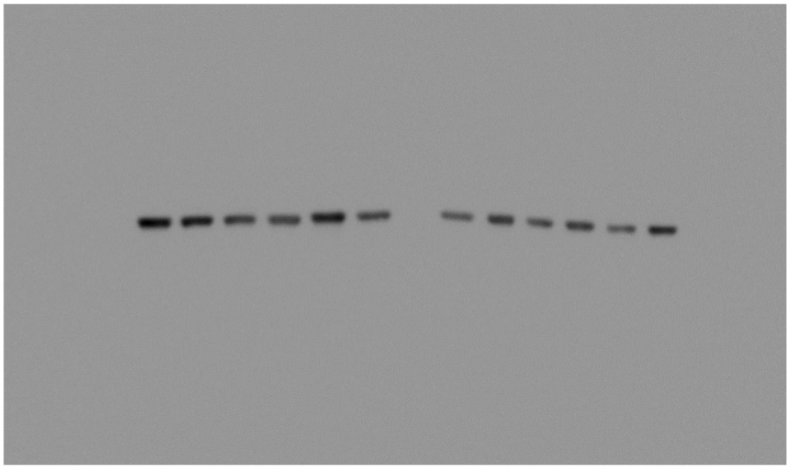

≈50 kDa

Figure S18 Full representative uncropped blots for SPAK and OXSR1 of images used in Figure 7

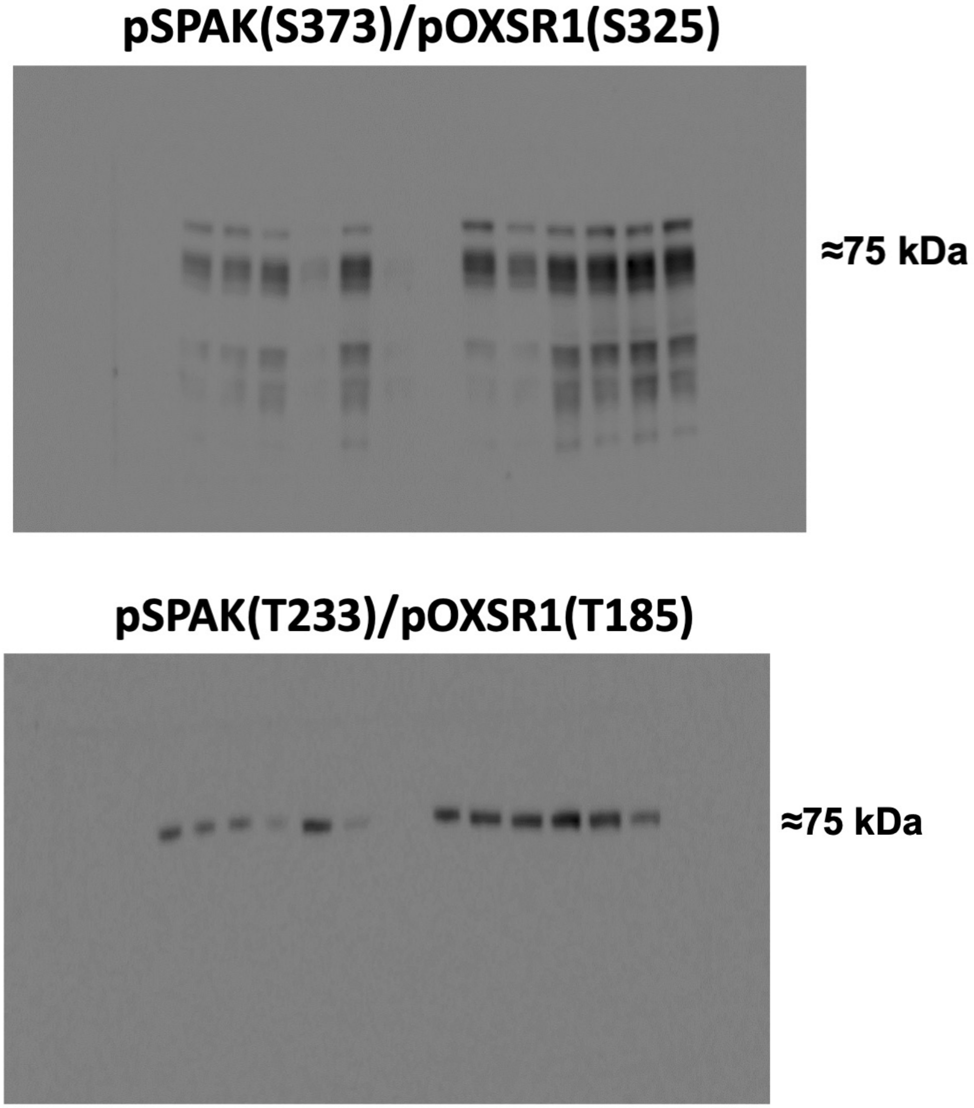

Figure S19 Full representative uncropped blots for pSPAK and pOXSR1 of images used in Figure 7

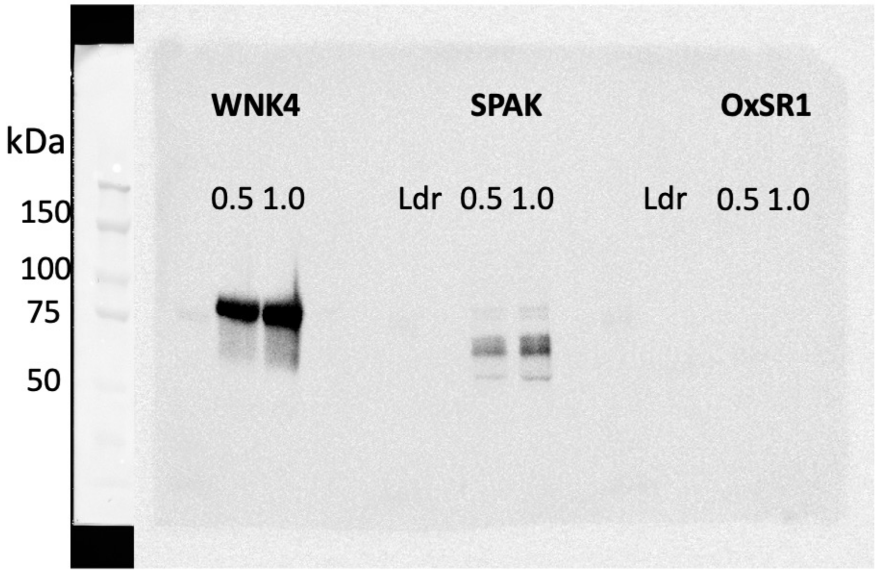

Figure S20 Immunoblot, with white light image of molecular weight marker included on the left for clarity, on which 0.5 and 1μg amounts of protein standards for WNK4, SPAK and OxSR1 loaded prior to immunoblotting for WNK4. As illustrated, we observe WNK4 at the expected molecular weight for this standard at approximately 77kDa and there is no cross reactivity with SPAK or OxSR1 standards at the expected molecular weight – with minor non-specific bands detected within the SPAK standard protein lanes. This figure is publicly available at https://figshare.com/articles/journal_contribution/AJP_Renal_online_supplement/23261495.

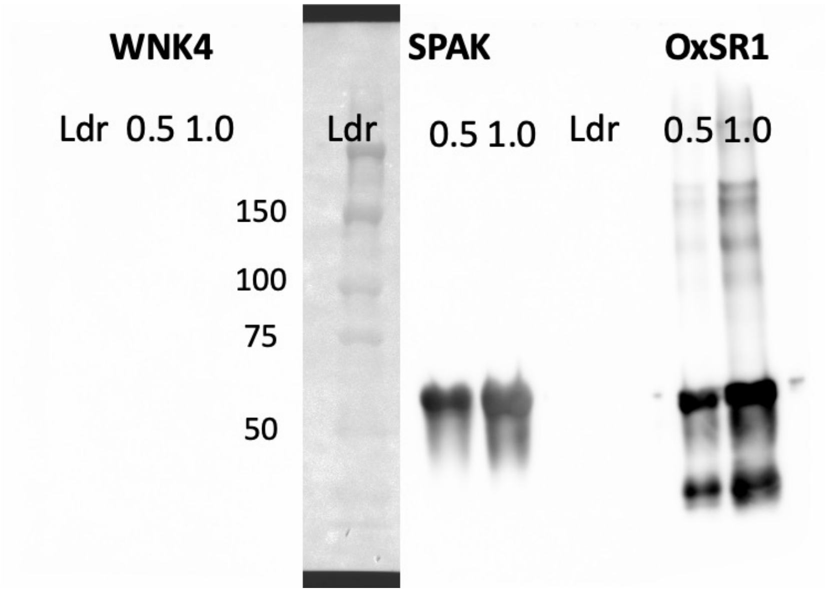

Figure S21 Immunoblot, with white light image of molecular weight marker included for clarity, on which 0.5 and 1μg amounts of protein standards for WNK4, SPAK and OxSR1 loaded prior to immunoblotting for SPAK. As illustrated, we observe SPAK at the expected molecular weight for this standard at approximately 63kDa and there is no cross reactivity with the WNK4 standard. We observe nonspecific bands above and below the expected SPAK signal with the OxSR1 standard. Further, as observed our rat kidney samples OxSR1 is observed at approximately 50kDa and there is no that is no cross reactivity observed at this weight. This figure is publicly available at https://figshare.com/articles/journal_contribution/AJP_Renal_online_supplement/23261495.

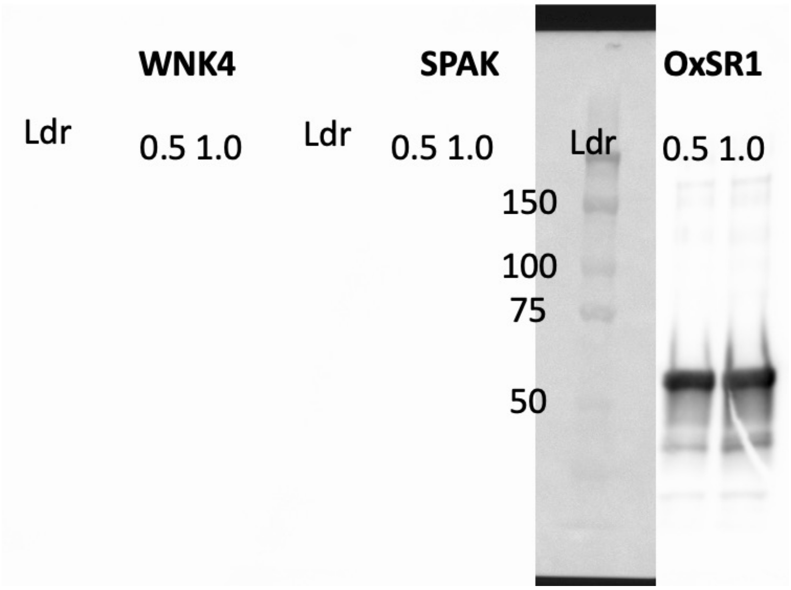

Figure S22 Immunoblot, with white light image of molecular weight marker included for clarity, on which 0.5 and 1μg amounts of protein standards for WNK4, SPAK and OxSR1 loaded prior to immunoblotting for OxSR1. As illustrated, we observe OxSR1 at the expected molecular weight for this standard at approximately 60kDa and there is no cross reactivity with SPAK or OxSR1 standards. This figure is publicly available at https://figshare.com/articles/journal_contribution/AJP_Renal_online_supplement/23261495.

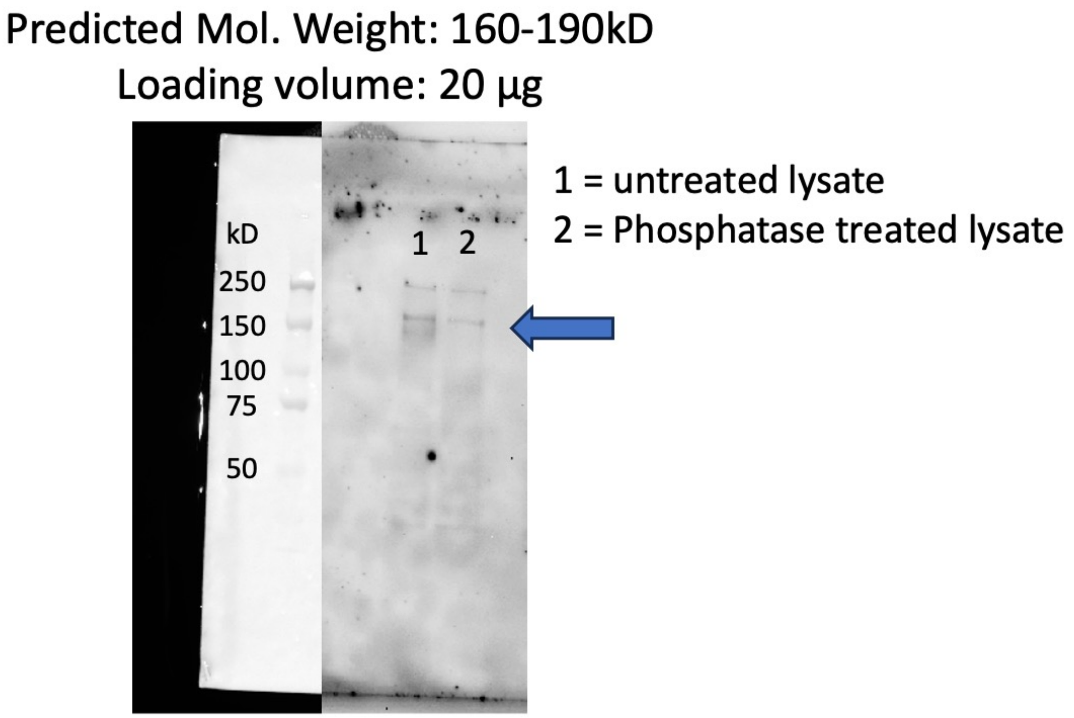

Figure S23 Representative blot, with white light image of molecular weight marker included on the left for clarity, illustrating significant loss of signal following sample incubation with phosphatase prior to immunoblotting with pNCC53 antibody that is detected at the expected molecular weight. This figure is publicly available at https://figshare.com/articles/journal_contribution/AJP_Renal_online_supplement/23261495.

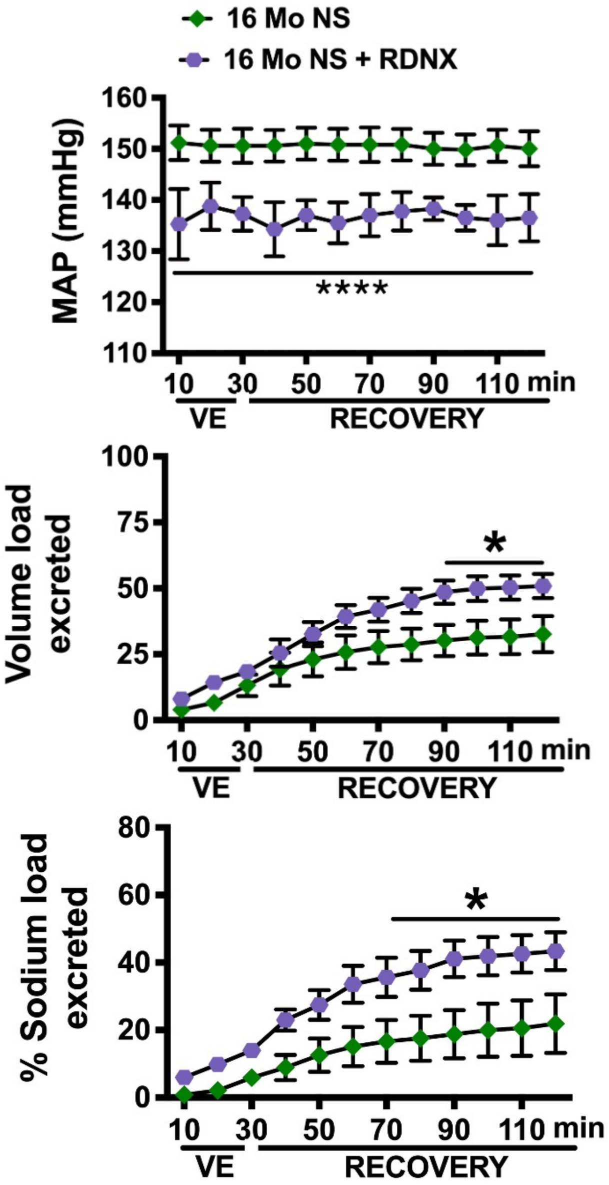

Figure S24 Mean arterial pressure (MAP; mmHg), urinary volume (% volume load excreted) and urinary sodium excretion (% sodium load excreted) in response to a 30-min isotonic saline volume expansion (VE) of 5% body weight followed by a 90-min recovery period in conscious 16-month-old male Sprague-Dawley rats and conscious 16-month-old male Sprague-Dawley rats that underwent bilateral renal denervation 10-14 days previously maintained on a lifelong normal salt intake (NS; 0.6% NaCl), N=6 per group mean ± SD. MAP = mean arterial pressure. *P< 0.05 vs. respective16-month-old group control value. Values for 16-month-old male rats are replicated from Figure S9 for comparison. Renal denervation efficacy was confirmed as a reduction in renal NE content below 10% of that observed in control 3-month-old rats.
